# Distribution and identification of the species in the genus *Helicops* Wagler, 1830 (Serpentes, Colubridae, Xenodontinae)

**DOI:** 10.3897/BDJ.10.e69234

**Published:** 2022-03-10

**Authors:** Yannis Schöneberg, Gunther Köhler

**Affiliations:** 1 Johann Wolfgang Goethe-University, Frankfurt am Main, Germany Johann Wolfgang Goethe-University Frankfurt am Main Germany; 2 Senckenberg Society for Nature Research, Frankfurt am Main, Germany Senckenberg Society for Nature Research Frankfurt am Main Germany

**Keywords:** annotated list, aquatic snakes, distribution maps, identification key, morphology, neotropics, pholidosis, taxonomy

## Abstract

**Background:**

The aquatic snakes of the genus *Helicops* are widely distributed throughout northern South America, but understudied concerning some aspects, including morphological traits and distribution. The most recent publication that provided an identification key to all species of *Helicops* is over 50 years old. This key is of limited value today since it includes taxa no longer recognised and lacks 8 of the 19 species currently recognised. There never was a publication trying to summarise distributional and morphological information of all species of *Helicops*. Most knowledge of these species is distributed throughout many small publications, such as short observation notes.

**New information:**

Here, we present distribution maps (point records), an identification key and comments on identification for all species in this genus. We base our results on a comprehensive literature review of over 300 scientific publications and own examinations. Our examinations comprise 190 specimens of 10 of the 19 currently recognised species and one *Helicops* sp. We report range extensions for the species *H.danieli*, *H.infrataeniatus*, *H.leopardinus*, *H.pastazae* and *H.polylepis*.

## Introduction

Water snakes of the genus *Helicops* are widely distributed, mainly in the northern half of South America, but they also reach Uruguay and central Argentina. The genus currently comprises 19 species of aquatic snakes inhabiting nearly all kinds of water bodies within their distribution range, from small ponds and puddles to slow-flowing streams, also in urban areas (França et al. 2012, Hernández-Ruiz et al. 2014, Koski et al. 2016). However, for many species, not much more than their description is known. The genus *Helicops* belongs to the Hydropsini tribe together with the genera *Hydrops* and *Pseudoeryx*. The Hydropsini are part of the subfamily Dipsadinae and characterised by the wide origin of the superficialis muscle (Di Pietro et al. 2014). All 19 species of the genus *Helicops* share the combination of having eyes and nostrils in a dorsal position on the top of the head, a single internasal scale, a divided cloacal shield and at least some keeled dorsal scales (Costa et al. 2016). The first description of the species in this genus was *Helicopsangulatus*, which was described as *Coluberangulatus* and *C.alidras* by Linnaeus (1758). In 1830, Wagler (1830) assigned *C.angulatus* and other species of the genera *Coluber* and *Natrix* to the newly-created genus *Helicops*. The last identification key was published by Peters and Orejas-Miranda (1970). In this work, the authors recognised 13 species, including the no longer recognised *H.hogei* (currently synonym of *H.scalaris*) and *H.pictiventris* (currently synonym of *H.infrataeniatus*) (Rossman 2002). Since then, *H.infrataeniatus* has been raised to species level again and seven new species have been described (*H.apiaka*, *H.boitata*, *H.nentur*, *H.phantasma*, *H.petersi*, *H.tapajonicus* and *H.yacu*). Of these, more than half were discovered in the past 10 years (*H.apiaka*, *H.boitata*, *H.nentur* and *H.phantasma*). For these seven new species, not much more than their description is known. This and the number of recently described new species show the missing taxonomic overview for this genus. Most of the knowledge is scattered across many publications. Especially distributional information is mostly presented in observation notes. Therefore, we aim to present a basis for further taxonomic studies, by providing point records, an annotated checklist and an identification key, based on a comprehensive literature review and own observations.

## Materials and methods

We base our species assessment of the genus *Helicops* on the morphological examination of 190 specimens, representing 10 of the 19 currently recognised species in this genus and on a comprehensive literature review. The examined specimens are located in six herpetological museum collections in Germany: Senckenberg Research Institution Frankfurt (SMF); Senckenberg Naturhistorische Sammlungen Dresden (MTKD); Zoologisches Forschungsmuseum Alexander König in Bonn (ZFMK); Zoologische Staatssammlung München (ZSM); Staatliches Museum für Naturkunde Stuttgart (SMNS) and Naturkundemuseum Berlin (NMB).

The examined morphologic characters were: snout-vent length (SVL), tail length (TL), the ratio between tail length and snout-vent length (TL/SVL), number of ventral shields (VE), number of subcaudal scales (SC), presence of subcaudal keels (SCK), number of preoculars (PRO), number of postoculars (PSO), number of loreals (LO), number of anterior temporals (AT), number of posterior temporals (PT), number of supralabials (SL), number of supralabials in contact with the eye (SL+E), number of infralabials (IL), number of dorsal scale rows at mid-body (DSM), presence of dorsal keels at mid-body (DKM), number of dorsal scale rows approximately a head length prior to cloaca (DSP), presence of dorsal keels approximately a head length prior to cloaca (DKP), if cloacal plate is divided (CL), if nasal scale is divided, semi-divided or entire (NA) and presence of intergenials (IG). For male and female specimens, we recorded the number of ventral scales, number of subcaudal scales, snout-vent length, tail length and the ratio between snout vent length and tail length separately. Measurements were taken using a millimetric tape measure and ventrals were counted as proposed by Dowling (1951). We determined the sex of each specimen by exterior examination of the shape of the tail base (tail base bulge caused by presence of hemipenes in males, bulging absent in females). The results of our examinations are available in Suppl. material 1. We did not take tail measurements in specimens with caudal damage. Head scutellation was recorded for each side separately.

We base the species’ distribution summaries on locality data of the examined specimens and additionally on literature data. One further locality was detected by browsing through iNaturalist (https://www.inaturalist.org/observations/9053312). We could identify the species (*H.polylepis*) by its unique colouration. Only records were included for which a reliable description of the locality was available, i.e. a map which enables the extraction of the coordinates or they were provided directly. In Nogueira et al. (2019), we were not able to unambiguously identify some of the cited literature, even with the help of the authors. Therefore, we simply pass their citation to our supplement with a respective note. We treat distribution records with ambiguous references separately in the distribution maps and supplements. All details on literature used and references for the distribution records are listed in Suppl. material 2.

We created the distribution maps using QGIS 3.12.2 and maps freely available at naturalearthdata.com.

We created the identification key using the morphological data gathered by examining specimens and literature data. Literature references used for morphology are listed in Suppl. material 3.

## Data resources

Suppl. materials 1, 2 contain tables in tab delimited text format. Suppl. material 1 contains the examination results for each specimen. It has columns with following headers: Species; Catalog numb.; Sex; Snout-venth length [mm]; Tail length [mm]; TL/SVL; Ventrals; Subcaudals; Subcaudal keels; Preoculars right; Preoculars left; Loreals right; Loreals left; Postoculars right; Postoculars left; Anterior Temporals right; Anterior Temporals left; Posterior Temporals right; Posterior Temporals left; Supralabials right; Supralabials left; Supralabials + Eye right; Supralabials + Eye left; Sublabials right; Sublabials left; Dorsal scale rows at mid-body; Dorsal keels at mid-body; Dorsal scale rows at posterior body; Dorsal keels at posterior body; Analplate; Intergenials. The content of the columns follows the description of the examined morphological characters in the Methods section. Suppl. material 2 contains all distribution records extracted from literature and their respective reference. It has columns with the following headers: Species; Country; Province; Locality; Locality notes; Latitude (DD); Longitude (DD); Latitude (DMS); Longitude (DMS); Literature; Online version. Most of the columns are self-explanatory. The columns Latitude (DD) and Longitude (DD) contain the coordinates in Decimal degree and the columns Latitude (DMS) and Longitude (DMS) contain the coordinates in Degrees Minutes Seconds format.

Suppl. material 3 is a plain text file containing all the references used for the morphological assessment. Each reference is provided in a separate line.

## Taxon treatments

### Helicops angulatus

(Linnaeus, 1758)

D8B64A07-3420-537D-BA38-929FB9493E89

#### Materials

- **Type status:** Other material. **Occurrence:** catalogNumber: MTKD 15294; recordedBy: Fritzsche leg.; individualCount: 1; sex: male; **Taxon:** scientificName: *Helicopsangulatus* (Linnaeus, 1758); **Location:** country: Brazil; **Record Level:** institutionID: MTKD
- **Type status:** Other material. **Occurrence:** catalogNumber: MTKD 15509; recordedBy: Poeppig leg.; individualCount: 1; sex: female; **Taxon:** scientificName: *Helicopsangulatus* (Linnaeus, 1758); **Location:** country: Brazil; stateProvince: unknown province; locality: Ega ad Amazonas; **Event:** year: 1831; **Record Level:** institutionID: MTKD
- **Type status:** Other material. **Occurrence:** catalogNumber: MTKD 41670; individualCount: 1; sex: female; **Taxon:** scientificName: *Helicopsangulatus* (Linnaeus, 1758); **Location:** country: Peru; **Record Level:** institutionID: MTKD
- **Type status:** Other material. **Occurrence:** catalogNumber: SMF 100016; recordedBy: M. Jansen leg.; individualCount: 1; sex: female; **Taxon:** scientificName: *Helicopsangulatus* (Linnaeus, 1758); **Location:** country: Bolivia; stateProvince: Santa Cruz; locality: Nuflo de Chavez, RPPN San Sebastian; verbatimLocality: 524; verbatimLatitude: -16°23.263; verbatimLongitude: -61°59.983; **Event:** year: 2006; **Record Level:** institutionID: SMF
- **Type status:** Other material. **Occurrence:** catalogNumber: SMF 17817; individualCount: 1; sex: female; **Taxon:** scientificName: *Helicopsangulatus* (Linnaeus, 1758); **Record Level:** institutionID: SMF
- **Type status:** Other material. **Occurrence:** catalogNumber: SMF 17818; individualCount: 1; sex: female; **Taxon:** scientificName: *Helicopsangulatus* (Linnaeus, 1758); **Record Level:** institutionID: SMF
- **Type status:** Other material. **Occurrence:** catalogNumber: SMF 17819; individualCount: 1; sex: female; **Taxon:** scientificName: *Helicopsangulatus* (Linnaeus, 1758); **Record Level:** institutionID: SMF
- **Type status:** Other material. **Occurrence:** catalogNumber: SMF 17820; individualCount: 1; sex: male; **Taxon:** scientificName: *Helicopsangulatus* (Linnaeus, 1758); **Record Level:** institutionID: SMF
- **Type status:** Other material. **Occurrence:** catalogNumber: SMF 32409; individualCount: 1; sex: female; **Taxon:** scientificName: *Helicopsangulatus* (Linnaeus, 1758); **Record Level:** institutionID: SMF
- **Type status:** Other material. **Occurrence:** catalogNumber: SMF 32410; individualCount: 1; sex: female; **Taxon:** scientificName: *Helicopsangulatus* (Linnaeus, 1758); **Record Level:** institutionID: SMF
- **Type status:** Other material. **Occurrence:** catalogNumber: SMF 40029; individualCount: 1; sex: female; **Taxon:** scientificName: *Helicopsangulatus* (Linnaeus, 1758); **Record Level:** institutionID: SMF
- **Type status:** Other material. **Occurrence:** catalogNumber: SMF 80033; recordedBy: Edgar Lehr leg.; individualCount: 1; sex: female; **Taxon:** scientificName: *Helicopsangulatus* (Linnaeus, 1758); **Location:** country: Peru; stateProvince: Ucayali; locality: Bolognesi (Campamento); verbatimLocality: 230; verbatimLatitude: -10°6.217; verbatimLongitude: -73°49.033; **Event:** year: 1998; **Record Level:** institutionID: SMF
- **Type status:** Other material. **Occurrence:** catalogNumber: SMF 90947; recordedBy: Gunther Köhler, R. Seipp, S. Moya leg.; individualCount: 1; sex: female; **Taxon:** scientificName: *Helicopsangulatus* (Linnaeus, 1758); **Location:** country: Ecuador; stateProvince: Pastaza; locality: Arutam, km 48 Transamazonica; verbatimLocality: 880; verbatimLatitude: -1°47.07; verbatimLongitude: -77°49.96; **Event:** year: 1996; **Record Level:** institutionID: SMF
- **Type status:** Other material. **Occurrence:** catalogNumber: SMF 91832; individualCount: 1; sex: male; **Taxon:** scientificName: *Helicopsangulatus* (Linnaeus, 1758); **Record Level:** institutionID: SMF
- **Type status:** Other material. **Occurrence:** catalogNumber: SMF 91833; individualCount: 1; sex: female; **Taxon:** scientificName: *Helicopsangulatus* (Linnaeus, 1758); **Record Level:** institutionID: SMF
- **Type status:** Other material. **Occurrence:** catalogNumber: SMF 91834; individualCount: 1; sex: female; **Taxon:** scientificName: *Helicopsangulatus* (Linnaeus, 1758); **Record Level:** institutionID: SMF
- **Type status:** Other material. **Occurrence:** catalogNumber: SMNS 13438; recordedBy: Hartmann leg.; individualCount: 1; sex: female; **Taxon:** scientificName: *Helicopsangulatus* (Linnaeus, 1758); **Location:** country: Suriname; **Event:** year: 1893; **Record Level:** institutionID: SMF
- **Type status:** Other material. **Occurrence:** catalogNumber: SMNS 3063; recordedBy: F. Glocker leg.; individualCount: 1; sex: female; **Taxon:** scientificName: *Helicopsangulatus* (Linnaeus, 1758); **Location:** country: Brazil; stateProvince: Bahia; **Event:** year: 1854; **Record Level:** institutionID: SMNS
- **Type status:** Other material. **Occurrence:** catalogNumber: SMNS 3064.1; recordedBy: A. Kappler; individualCount: 1; sex: female; **Taxon:** scientificName: *Helicopsangulatus* (Linnaeus, 1758); **Event:** year: 1843; **Record Level:** institutionID: SMNS
- **Type status:** Other material. **Occurrence:** catalogNumber: SMNS 3064.2; individualCount: 1; sex: male; **Taxon:** scientificName: *Helicopsangulatus* (Linnaeus, 1758); **Record Level:** institutionID: SMNS
- **Type status:** Other material. **Occurrence:** catalogNumber: SMNS 6394; recordedBy: A. Schlüter leg.; individualCount: 1; sex: male; **Taxon:** scientificName: *Helicopsangulatus* (Linnaeus, 1758); **Event:** year: 1985; **Record Level:** institutionID: SMNS
- **Type status:** Other material. **Occurrence:** catalogNumber: ZFMK 47670; individualCount: 1; sex: female; **Taxon:** scientificName: *Helicopsangulatus* (Linnaeus, 1758); **Location:** country: Guyana; stateProvince: unknown province; locality: Roraima-Gebiet; **Record Level:** institutionID: ZFMK
- **Type status:** Other material. **Occurrence:** catalogNumber: ZFMK 8403; individualCount: 1; sex: male; **Taxon:** scientificName: *Helicopsangulatus* (Linnaeus, 1758); **Location:** country: Brazil; stateProvince: Amazonas; locality: Jurua; **Record Level:** institutionID: ZFMK
- **Type status:** Other material. **Occurrence:** catalogNumber: ZFMK 8404; individualCount: 1; sex: female; **Taxon:** scientificName: *Helicopsangulatus* (Linnaeus, 1758); **Location:** country: Brazil; stateProvince: Amazonas; locality: Jurua; **Record Level:** institutionID: ZFMK
- **Type status:** Other material. **Occurrence:** catalogNumber: ZMB 10854; recordedBy: S. Eye leg.; individualCount: 1; sex: male; **Taxon:** scientificName: *Helicopsangulatus* (Linnaeus, 1758); **Location:** country: Brazil; stateProvince: Maranhao; **Record Level:** institutionID: ZMB
- **Type status:** Other material. **Occurrence:** catalogNumber: ZMB 2303; recordedBy: M. Bloch leg.; individualCount: 1; sex: female; **Taxon:** scientificName: *Helicopsangulatus* (Linnaeus, 1758); **Record Level:** institutionID: ZMB
- **Type status:** Other material. **Occurrence:** catalogNumber: ZMB 2305; individualCount: 1; sex: female; **Taxon:** scientificName: *Helicopsangulatus* (Linnaeus, 1758); **Location:** country: French Guiana; **Record Level:** institutionID: ZMB
- **Type status:** Other material. **Occurrence:** catalogNumber: ZMB 25975A; recordedBy: K. Heller leg.; individualCount: 1; sex: female; **Taxon:** scientificName: *Helicopsangulatus* (Linnaeus, 1758); **Location:** country: Suriname; stateProvince: Paramaribo; locality: Paramaribo; **Record Level:** institutionID: ZMB
- **Type status:** Other material. **Occurrence:** catalogNumber: ZMB 25975B; recordedBy: K. Heller leg.; individualCount: 1; sex: male; **Taxon:** scientificName: *Helicopsangulatus* (Linnaeus, 1758); **Location:** country: Suriname; stateProvince: Paramaribo; locality: Paramaribo; **Record Level:** institutionID: ZMB
- **Type status:** Other material. **Occurrence:** catalogNumber: ZMB 26382; recordedBy: K. Heller leg.; individualCount: 1; sex: male; **Taxon:** scientificName: *Helicopsangulatus* (Linnaeus, 1758); **Location:** country: Suriname; stateProvince: Paramaribo; locality: Paramaribo; **Record Level:** institutionID: ZMB
- **Type status:** Other material. **Occurrence:** catalogNumber: ZMB 27783; recordedBy: Aq. Zoo. leg.; individualCount: 1; sex: male; **Taxon:** scientificName: *Helicopsangulatus* (Linnaeus, 1758); **Location:** country: Brazil; **Record Level:** institutionID: ZMB
- **Type status:** Other material. **Occurrence:** catalogNumber: ZMB 47771; recordedBy: K. Lako leg.; individualCount: 1; sex: male; **Taxon:** scientificName: *Helicopsangulatus* (Linnaeus, 1758); **Location:** country: Brazil; stateProvince: Para; locality: Rio Caramarapy; **Record Level:** institutionID: ZMB
- **Type status:** Other material. **Occurrence:** catalogNumber: ZMB 54167; individualCount: 1; sex: female; **Taxon:** scientificName: *Helicopsangulatus* (Linnaeus, 1758); **Location:** country: French Guiana; **Record Level:** institutionID: ZMB
- **Type status:** Other material. **Occurrence:** catalogNumber: ZMB 64697; recordedBy: Anat. Sammlung leg.; individualCount: 1; sex: female; **Taxon:** scientificName: *Helicopsangulatus* (Linnaeus, 1758); **Record Level:** institutionID: ZMB
- **Type status:** Other material. **Occurrence:** catalogNumber: ZMB 64698; recordedBy: Anat. Sammlung leg.; individualCount: 1; sex: female; **Taxon:** scientificName: *Helicopsangulatus* (Linnaeus, 1758); **Record Level:** institutionID: ZMB
- **Type status:** Other material. **Occurrence:** catalogNumber: ZMB 89648; individualCount: 1; sex: female; **Taxon:** scientificName: *Helicopsangulatus* (Linnaeus, 1758); **Record Level:** institutionID: ZMB
- **Type status:** Other material. **Occurrence:** catalogNumber: ZMB 89649; individualCount: 1; sex: female; **Taxon:** scientificName: *Helicopsangulatus* (Linnaeus, 1758); **Record Level:** institutionID: ZMB
- **Type status:** Other material. **Occurrence:** catalogNumber: ZSM 1525/0; individualCount: 1; sex: male; **Taxon:** scientificName: *Helicopsangulatus* (Linnaeus, 1758); **Location:** country: Suriname; **Event:** year: <1858; **Record Level:** institutionID: ZSM
- **Type status:** Other material. **Occurrence:** catalogNumber: ZSM 1526/0; individualCount: 1; sex: male; **Taxon:** scientificName: *Helicopsangulatus* (Linnaeus, 1758); **Location:** country: Brazil; **Event:** year: <1907; **Record Level:** institutionID: ZSM
- **Type status:** Other material. **Occurrence:** catalogNumber: ZSM 1528/0; recordedBy: Spix & Martius leg.; individualCount: 1; sex: male; **Taxon:** scientificName: *Helicopsangulatus* (Linnaeus, 1758); **Location:** country: Brazil; locality: in provinciae Bahiae adjacentibus; **Event:** year: 1817-1820; **Record Level:** institutionID: ZSM
- **Type status:** Other material. **Occurrence:** catalogNumber: ZSM 247/1983; recordedBy: L. Müller leg.; individualCount: 1; sex: female; **Taxon:** scientificName: *Helicopsangulatus* (Linnaeus, 1758); **Location:** country: Brazil; stateProvince: Pará; locality: Peixeboi (an der Bragançabahn); **Event:** year: 1910; **Record Level:** institutionID: ZSM
- **Type status:** Other material. **Occurrence:** catalogNumber: ZSM 264/2017; recordedBy: E. Snethlage leg.; individualCount: 1; sex: female; **Taxon:** scientificName: *Helicopsangulatus* (Linnaeus, 1758); **Location:** country: Brazil; stateProvince: Pará; locality: Rio Branco bei Obidos; **Event:** year: 1912; **Record Level:** institutionID: ZSM
- **Type status:** Other material. **Occurrence:** catalogNumber: ZSM 37/2015; recordedBy: E.-G. Burmeister leg.; individualCount: 1; sex: female; **Taxon:** scientificName: *Helicopsangulatus* (Linnaeus, 1758); **Location:** country: Peru; stateProvince: Huánuco; locality: Biologische Station Panguana (unterer Rio Yuyapichis, ca. 140 km SSW Pucallpa); **Event:** year: 1982; **Record Level:** institutionID: ZSM
- **Type status:** Other material. **Occurrence:** catalogNumber: ZSM 516/2003; recordedBy: R. Zischka leg.; individualCount: 1; sex: male; **Taxon:** scientificName: *Helicopsangulatus* (Linnaeus, 1758); **Location:** country: Bolivia; stateProvince: Chapare; **Event:** year: 1949; **Record Level:** institutionID: ZSM
- **Type status:** Other material. **Occurrence:** catalogNumber: ZSM 518/2003; recordedBy: H. Herkner leg.; individualCount: 1; sex: male; **Taxon:** scientificName: *Helicopsangulatus* (Linnaeus, 1758); **Location:** country: Columbia; stateProvince: Guainía; locality: Inirida; **Event:** year: 1976; **Record Level:** institutionID: ZSM
- **Type status:** Other material. **Occurrence:** catalogNumber: ZSM 59/1985; recordedBy: Maulhardt leg.; individualCount: 1; sex: female; **Taxon:** scientificName: *Helicopsangulatus* (Linnaeus, 1758); **Location:** country: Peru; stateProvince: Ucayali; locality: Yarina Cocha bei Pucallpa; **Event:** year: 1982; **Record Level:** institutionID: ZSM
- **Type status:** Other material. **Occurrence:** catalogNumber: ZSM 595/2003; recordedBy: O. Schindler leg.; individualCount: 1; sex: male; **Taxon:** scientificName: *Helicopsangulatus* (Linnaeus, 1758); **Location:** country: Brazil; stateProvince: Sao Paulo; locality: Puerto Tiberica am Rio Parana [= Porto Tibirica]; **Event:** year: 1938; **Record Level:** institutionID: ZSM

#### Diagnosis

*Helicopsangulatus* can be distinguished from all its congeners, except *H.scalaris* and *H.apiaka* by having subcaudal keels, 17–20 dorsal scale rows at mid-body (compared to 19 in *H.gomesi*; 21–27 in *H.hagmanni*; 23–25 in *H.pastazae*) and 103–123 ventrals (compared to 125–132 in *H.gomesi*; 117–138 in *H.hagmanni*). From *H.scalaris*, it differs in having no intergenials (for information on references, see Suppl. material 3; for summarised pholidosis information of the examined specimens, see Tables 1, 2). For differentiation from *H.apiaka*, see identification of *H.apiaka*.

#### Distribution

The distribution of *H.angulatus* extends over nearly the complete northern part of South America. As shown in Fig. 1a, the distribution range extends from Columbia to the east coast of Brazil and from Venezuela and offshore islands to the Brazilian Province of Sao Paulo and Bolivia.

### Helicops apiaka

Kawashita-Ribeiro, Àvila, Morais, 2013

A770EC5F-DEE2-5486-95F2-75FA9C869985

#### Diagnosis

According to the information given in Kawashita-Ribeiro et al. (2013), *H.apiaka* can be distinguished from all its congeners, except *H.angulatus* by the following combination of characteristics: absent intergenials, subcaudal keels present and 21–22 dorsal scale rows at mid-body (compared to 19 in *H.gomesi*). From *H.angulatus*, it differs by having 21–24 dorsal scale rows at anterior body, 21–22 at mid-body and 17–19 at posterior body (versus 19–21/19–20/17–19 in *H.angulatus*) and by having 118–127 ventral scales in males and 124–132 in females (versus 105–123 in male and 109–123 in female *H.angulatus*), as well as hemipenes morphology. The specimens examined in Kawashita-Ribeiro et al. (2013) originated from neighbouring areas to the *H.apiaka* locations. Our own examinations revealed that males of *H.angulatus* possess 103–119 ventrals in males and 104–125 in females. There is considerable overlap, especially between female specimens of the two species, thus excluding this character for identification. Besides that, we detected two specimens displaying morphology characters of *H.angulatus* (colouration, subcaudal keels, remaining pholidosis), but showing considerably more ventrals than other specimens of *H.angulatus* (SMF 17819, a female, with 156 ventrals and ZSM 0595/2003, female, with 130 ventrals). There is no locality information available for SMF 17819. ZSM 0595/2003 was collected at the Rio Parana in Porto Tibiriça, Sao Paulo, Brazil. This is approximately 1300 km distant from the distribution range of *H.apiaka*. The origin of a speciemen is probably an important feature in order to assign it to one of the two species (for information on references, see Suppl. material 3).

#### Distribution

The only known specimens of *H.apiaka* are from northern Mato Grosso and southern Pará (Fig. 1b).

#### Morphology remark

Regarding the number of dorsal scale roles at mid-body in *H.apiaka*, there is contradictory information. Moraes-da-Silva et al. (2019) states that *H.apiaka* has 19–21 dorsal scale rows at mid-body, which would eliminate this character as a diagnostic character to distinguish it from *H.angulatus*. This would leave only the number of ventrals in females as a sure diagnostic trait. However, in the original species description, Kawashita-Ribeiro et al. (2013) report 21–22 scale rows. At this point, we trust the data given by Kawashita-Ribeiro et al. (2013), as both publications examined the same specimens.

### Helicops boitata

Moraes-da-Silva, Amaro, Sales-Nunes, Strüssmann, Teixeira, Andrade, Sudré, Recoder, Rodrigues, Curcio, 2019

B8DBA002-28F0-528E-80B9-03FFAEE9B181

#### Diagnosis

*Helicopsboitata* differs from all its congeners by the combination of an entire nasal scale and 25 dorsal scale rows at mid-body, reducing to 21 anterior to cloaca (versus 17/15 in *H.nentur*; 28–28/18–20 in *H.yacu*) (for information on references, see Suppl. material 3).

#### Distribution

*Helicopsboitata* is only known from the Pantanal at Transpantaneira Road in the Municipality of Pocone, Mato Grosso, Brazil (Fig. 1c).

### Helicops carinicaudus

(Wied–Neuwied, 1825)

8E1C4D52-0AA8-5945-9988-0122F9469806

#### Materials

- **Type status:** Other material. **Occurrence:** catalogNumber: MTKD 15295; recordedBy: Fritzsche leg.; individualCount: 1; sex: male; **Taxon:** scientificName: *Helicopscarinicaudus* (Wied–Neuwied, 1825); **Location:** country: Brazil; **Record Level:** institutionID: MTKD
- **Type status:** Other material. **Occurrence:** catalogNumber: MTKD 15505; individualCount: 1; sex: male; **Taxon:** scientificName: *Helicopscarinicaudus* (Wied–Neuwied, 1825); **Location:** country: Brazil; **Record Level:** institutionID: MTKD
- **Type status:** Other material. **Occurrence:** catalogNumber: SMF 17799; recordedBy: H. Ihering leg.; individualCount: 1; sex: female; **Taxon:** scientificName: *Helicopscarinicaudus* (Wied–Neuwied, 1825); **Location:** country: Brazil; stateProvince: Rio Grande do Sul; locality: Rio Grande; **Event:** year: 1886; **Record Level:** institutionID: SMF
- **Type status:** Other material. **Occurrence:** catalogNumber: SMF 17800; recordedBy: C.v.Heyden leg.; individualCount: 1; sex: male; **Taxon:** scientificName: *Helicopscarinicaudus* (Wied–Neuwied, 1825); **Location:** country: Brazil; **Event:** year: 1838; **Record Level:** institutionID: SMF
- **Type status:** Other material. **Occurrence:** catalogNumber: SMF 34035; recordedBy: E. Bresslau leg.; individualCount: 1; sex: female; **Taxon:** scientificName: *Helicopscarinicaudus* (Wied–Neuwied, 1825); **Location:** country: Brazil; stateProvince: Pernambuco; **Event:** year: 1913-1914; **Record Level:** institutionID: SMF
- **Type status:** Other material. **Occurrence:** catalogNumber: SMF 37925; recordedBy: A. Adolff leg.; individualCount: 1; sex: female; **Taxon:** scientificName: *Helicopscarinicaudus* (Wied–Neuwied, 1825); **Location:** country: Brazil; stateProvince: Rio Grande do Sul; locality: Porto Alegre; **Event:** year: 1935; **Record Level:** institutionID: SMF
- **Type status:** Other material. **Occurrence:** catalogNumber: SMF 49723; recordedBy: M. Schetty leg.; individualCount: 1; sex: male; **Taxon:** scientificName: *Helicopscarinicaudus* (Wied–Neuwied, 1825); **Location:** country: Brazil; stateProvince: Sao Paulo; locality: Umgebung von Sao Paulo; **Event:** year: 1955; **Record Level:** institutionID: SMF
- **Type status:** Other material. **Occurrence:** catalogNumber: SMF 51208; recordedBy: R. Mertens leg.; individualCount: 1; sex: female; **Taxon:** scientificName: *Helicopscarinicaudus* (Wied–Neuwied, 1825); **Location:** country: Brazil; stateProvince: Sao Paulo; locality: Rinopolis; **Event:** year: 1954; **Record Level:** institutionID: SMF
- **Type status:** Other material. **Occurrence:** catalogNumber: ZFMK 30350; individualCount: 1; sex: female; **Taxon:** scientificName: *Helicopscarinicaudus* (Wied–Neuwied, 1825); **Location:** country: Argentina; stateProvince: Buenos Aires; locality: Punta Lara; **Record Level:** institutionID: ZFMK
- **Type status:** Other material. **Occurrence:** catalogNumber: ZMB 2296; individualCount: 1; sex: female; **Taxon:** scientificName: *Helicopscarinicaudus* (Wied–Neuwied, 1825); **Record Level:** institutionID: ZMB
- **Type status:** Other material. **Occurrence:** catalogNumber: ZMB 2298; recordedBy: I. v. Olfers leg.; individualCount: 1; sex: female; **Taxon:** scientificName: *Helicopscarinicaudus* (Wied–Neuwied, 1825); **Location:** country: Brazil; **Record Level:** institutionID: ZMB
- **Type status:** Other material. **Occurrence:** catalogNumber: ZSM 2585/0; individualCount: 1; sex: female; **Taxon:** scientificName: *Helicopscarinicaudus* (Wied–Neuwied, 1825); **Location:** country: Brazil; **Event:** year: <1907; **Record Level:** institutionID: ZSM

#### Diagnosis

*Helicopscarinicaudus* can be distinguished from all its congeners, except *H.danieli*, *H.infrataeniatus*, *H.leopardinus* and *H.phantasma* by the following combination of characteristics: 17–19 dorsal scale rows at mid-body, reducing to 17 anterior to cloaca (versus 17–20/17–19 in *H.angulatus*; 21–22/17–19 in *H.apiaka*; 25/21 in *H.boitata*; 19/19 in *H.gomesi*; 21–29/19–23 in *H.hagmanni*; 17–20/15–19 in *H.modestus*; 17/15 in *H.nentur*; 23–25/16–19 in *H.pastazae*; 21–23/16 in *H.petersi*; 23–26/17–21 in *H.polylepis*; 19–21/16–19 in *H.scalaris*; 19/17 in *H.tapajonicus*; 20–23/16–19 in *H.trivittatus*; 25–28/18–20 in *H.yacu*), 128–141 ventrals in males and 128–148 ventrals in females (versus ♂103–123♀104–125 in *H.angulatus*; ♂112–125♀112–124 in *H.modestus*; ♂110–119♀113–125 in *H.scalaris*; ♂118♀121–123 in *H.tapajonicus*). From *H.danieli* and *H.leopardinus*, it differs in having a striped or uniform dorsum versus blotched pattern in *H.danieli* and *H.leopardinus*. From *H.infrataeniatus* and *H.phantasma*, it differs in having a yellow or cream venter with two series of black semi-lunar marks, between these small, irregular black spots (for information on references, see Suppl. material 3).

#### Distribution

The distribution of *H.carinicaudus* extends from the Estuary of the Rio de La Plata along the shoreline of Brazil to the Province Pernambuco (Fig. 1d).

### Helicops danieli

Amaral, 1937

2F12CBD5-FE4C-5A5B-80F5-43458759C501

#### Materials

- **Type status:** Other material. **Occurrence:** catalogNumber: SMF 55074; recordedBy: A. Werner leg.; individualCount: 1; sex: female; **Taxon:** scientificName: *Helicopsdanieli* Amaral, 1937; **Location:** country: Columbia; stateProvince: Barranquilla; **Event:** year: 1958; **Record Level:** institutionID: SMF
- **Type status:** Other material. **Occurrence:** catalogNumber: SMF 55115; recordedBy: A. Werner leg.; individualCount: 1; sex: female; **Taxon:** scientificName: *Helicopsdanieli* Amaral, 1937; **Location:** country: Columbia; stateProvince: Barranquilla; **Event:** year: 1958; **Record Level:** institutionID: SMF
- **Type status:** Other material. **Occurrence:** catalogNumber: SMF 55695; recordedBy: A. Werner leg.; individualCount: 1; sex: female; **Taxon:** scientificName: *Helicopsdanieli* Amaral, 1937; **Location:** country: Columbia; stateProvince: Barranquilla; **Event:** year: 1958; **Record Level:** institutionID: SMF
- **Type status:** Other material. **Occurrence:** catalogNumber: ZMB 9490; individualCount: 1; sex: male; **Taxon:** scientificName: *Helicopsdanieli* Amaral, 1937; **Location:** country: Brazil; **Record Level:** institutionID: ZMB
- **Type status:** Other material. **Occurrence:** catalogNumber: ZSM 596/2003; recordedBy: W. Hellmich leg.; individualCount: 1; sex: female; **Taxon:** scientificName: *Helicopsdanieli* Amaral, 1937; **Location:** country: Columbia; stateProvince: Bolivar; locality: Jesus del Rio; **Event:** year: 1937; **Record Level:** institutionID: ZSM

#### Diagnosis

*Helicopsdanieli* is readily distinguished from its congeners by its unique colour pattern, namely a spotted dorsum in combination with a ventral pattern consisting of two rows of semi-lunar marks on a light background (for information on references see, Suppl. material 3).

#### Distribution

*Helicopsdanieli* is only occurring in Colombia, where it seems to be found mainly west of the Andes. There is a report from the lowland in the east near the Brazilian border (Yuki and Castano 1998, Fig. 1e). Specimen ZMB 9490 has the country-level locality Brazil without further precision.

### Helicops gomesi

Amaral, 1921

A147D389-F838-5614-9B40-3844E7BE46F9

#### Diagnosis

*Helicopsgomesi* is distinguished from all its congeners, except *H.angulatus* by having subcaudal keels, no intergenials, and 19 dorsal scale rows throughout its body (compared to 21–24/21–22/17–19 in *H.apiaka*). From *H.angulatus*, it differs in having 125–132 ventrals in males and 128–132 in females (versus 103–119 in males and 104–125 in females of *H.angulatus)*; (for information on references, see Suppl. material 3).

#### Distribution

The distribution of *H.gomesi* extends from the Brazilian Province Sao Paulo to the Provinces Mato Grosso, Mato Grosso do Sul and Goias (Fig. 1f).

### Helicops hagmanni

Roux, 1910

4B1DFB09-EE66-5BB2-AF50-BB9DA4C2D0A6

#### Materials

- **Type status:** Other material. **Occurrence:** catalogNumber: MTKD 7801; recordedBy: Poeppig leg.; individualCount: 1; sex: male; **Taxon:** scientificName: *Helicopshagmanni* Roux, 1910; **Location:** country: Brazil; stateProvince: Amazonas; **Event:** year: 1831; **Record Level:** institutionID: MTKD
- **Type status:** Other material. **Occurrence:** catalogNumber: ZMB C-826; recordedBy: A. Freiherr v. Dungern leg.; individualCount: 1; sex: female; **Taxon:** scientificName: *Helicopshagmanni* Roux, 1910; **Location:** country: Brazil; stateProvince: Para; locality: Umgebung von Para; **Record Level:** institutionID: ZMB

#### Diagnosis

*Helicopshagmanni* is distinguished from all its congeners by having subcaudal keels, 21–29 dorsal scale rows at mid-body (versus 17–20 in *H.angulatus*; 21–22 in *H.apiaka*; 19 in *H.gomesi*; 23–25 in *H.pastazae*) and 50–59 subcaudals (compared to 79–103 in *H.apiaka*; 72–117 in *H.pastazae*); (for information on references, see Suppl. material 3).

#### Distribution

The distribution of *H.hagmanni* ranges from the Estuary of the Amazonas to the Brazilian Provinces Amazonas, Acre, Rondônia and the Venezuelan Province Amazonas. There is also one record from south-western Colombia (Rossman 1975, Fig. 2a).

#### Morphology remark

The examined specimens had smooth subcaudal scales on the anterior part of the tail, changing to weakly-keeled scales at the posterior tail, which contrasts with the examination results in Moraes-da-Silva et al. (2019) who reported them to be absent, without further notes on methodology for this character (see also Tables 1, 2).

### Helicops infrataeniatus

Jan, 1865

FAC174B8-945D-5ED6-B3E2-AAEF8097F8EB

#### Materials

- **Type status:** Other material. **Occurrence:** catalogNumber: MTKD 29826; recordedBy: Strauss-Hiller leg.; individualCount: 1; sex: female; **Taxon:** scientificName: *Helicopsinfrataeniatus* Jan, 1865; **Location:** country: Argentina; stateProvince: Entre Rios; **Record Level:** institutionID: MTKD
- **Type status:** Other material. **Occurrence:** catalogNumber: SMF 17795; recordedBy: P. Werner leg.; individualCount: 1; sex: female; **Taxon:** scientificName: *Helicopsinfrataeniatus* Jan, 1865; **Location:** country: Brazil; **Event:** year: 1898; **Record Level:** institutionID: SMF
- **Type status:** Other material. **Occurrence:** catalogNumber: SMF 17796; recordedBy: A. Haas leg.; individualCount: 1; sex: female; **Taxon:** scientificName: *Helicopsinfrataeniatus* Jan, 1865; **Location:** country: Brazil; stateProvince: Parana; locality: Curityba; **Event:** year: 1905; **Record Level:** institutionID: SMF
- **Type status:** Other material. **Occurrence:** catalogNumber: SMF 17797; recordedBy: A. Haas leg.; individualCount: 1; sex: female; **Taxon:** scientificName: *Helicopsinfrataeniatus* Jan, 1865; **Location:** country: Brazil; stateProvince: Parana; locality: Curityba; **Event:** year: 1905; **Record Level:** institutionID: SMF
- **Type status:** Other material. **Occurrence:** catalogNumber: SMF 17801; recordedBy: H. Ihering leg.; individualCount: 1; sex: female; **Taxon:** scientificName: *Helicopsinfrataeniatus* Jan, 1865; **Location:** country: Brazil; stateProvince: Rio Grande do Sul; **Event:** year: 1888; **Record Level:** institutionID: SMF
- **Type status:** Other material. **Occurrence:** catalogNumber: SMF 51209; recordedBy: R. Mertens leg.; individualCount: 1; sex: male; **Taxon:** scientificName: *Helicopsinfrataeniatus* Jan, 1865; **Location:** country: Brazil; stateProvince: Rio Grande do Sul; locality: Espumoso Via Carasinho; **Event:** year: 1954; **Record Level:** institutionID: SMF
- **Type status:** Other material. **Occurrence:** catalogNumber: SMF 51210; recordedBy: R. Mertens leg.; individualCount: 1; sex: female; **Taxon:** scientificName: *Helicopsinfrataeniatus* Jan, 1865; **Location:** country: Brazil; stateProvince: Rio Grande do Sul; locality: Espumoso Via Carasinho; **Event:** year: 1954; **Record Level:** institutionID: SMF
- **Type status:** Other material. **Occurrence:** catalogNumber: SMF 67327; recordedBy: Foerster leg.; individualCount: 1; sex: male; **Taxon:** scientificName: *Helicopsinfrataeniatus* Jan, 1865; **Location:** country: Argentina; stateProvince: Chaco; locality: Roque Saenz Pena; **Event:** year: 1965; **Record Level:** institutionID: SMF
- **Type status:** Other material. **Occurrence:** catalogNumber: SMNS 3065; recordedBy: Umlauff leg.; individualCount: 1; sex: female; **Taxon:** scientificName: *Helicopsinfrataeniatus* Jan, 1865; **Location:** country: Brazil; **Event:** year: 1889; **Record Level:** institutionID: SMNS
- **Type status:** Other material. **Occurrence:** catalogNumber: SMNS 9038; recordedBy: A. Kwet leg.; individualCount: 1; sex: female; **Taxon:** scientificName: *Helicopsinfrataeniatus* Jan, 1865; **Location:** country: Brazil; stateProvince: Rio Grande do Sul; locality: Cachoeira do Sul; **Event:** year: 1995; **Record Level:** institutionID: SMNS
- **Type status:** Other material. **Occurrence:** catalogNumber: ZFMK 102469; recordedBy: leg.; individualCount: 1; sex: female; **Taxon:** scientificName: *Helicopsinfrataeniatus* Jan, 1865; **Location:** country: Brazil; stateProvince: Rio Grande do Sul; locality: Sao Leopoldo; **Record Level:** institutionID: SMNS
- **Type status:** Other material. **Occurrence:** catalogNumber: ZFMK 102499; individualCount: 1; sex: female; **Taxon:** scientificName: *Helicopsinfrataeniatus* Jan, 1865; **Location:** country: Argentina; stateProvince: Buenos Aires; locality: Punta Lara; **Record Level:** institutionID: ZFMK
- **Type status:** Other material. **Occurrence:** catalogNumber: ZFMK 102500; individualCount: 1; sex: female; **Taxon:** scientificName: *Helicopsinfrataeniatus* Jan, 1865; **Record Level:** institutionID: ZFMK
- **Type status:** Other material. **Occurrence:** catalogNumber: ZFMK 102501; individualCount: 1; sex: female; **Taxon:** scientificName: *Helicopsinfrataeniatus* Jan, 1865; **Record Level:** institutionID: ZFMK
- **Type status:** Other material. **Occurrence:** catalogNumber: ZFMK 102502; individualCount: 1; sex: female; **Taxon:** scientificName: *Helicopsinfrataeniatus* Jan, 1865; **Record Level:** institutionID: ZFMK
- **Type status:** Other material. **Occurrence:** catalogNumber: ZFMK 102503; individualCount: 1; sex: male; **Taxon:** scientificName: *Helicopsinfrataeniatus* Jan, 1865; **Record Level:** institutionID: ZFMK
- **Type status:** Other material. **Occurrence:** catalogNumber: ZFMK 102504; individualCount: 1; sex: female; **Taxon:** scientificName: *Helicopsinfrataeniatus* Jan, 1865; **Record Level:** institutionID: ZFMK
- **Type status:** Other material. **Occurrence:** catalogNumber: ZFMK 102505; individualCount: 1; sex: male; **Taxon:** scientificName: *Helicopsinfrataeniatus* Jan, 1865; **Record Level:** institutionID: ZFMK
- **Type status:** Other material. **Occurrence:** catalogNumber: ZFMK 102506; individualCount: 1; sex: female; **Taxon:** scientificName: *Helicopsinfrataeniatus* Jan, 1865; **Record Level:** institutionID: ZFMK
- **Type status:** Other material. **Occurrence:** catalogNumber: ZFMK 102507; individualCount: 1; sex: female; **Taxon:** scientificName: *Helicopsinfrataeniatus* Jan, 1865; **Record Level:** institutionID: ZFMK
- **Type status:** Other material. **Occurrence:** catalogNumber: ZFMK 102508; individualCount: 1; sex: female; **Taxon:** scientificName: *Helicopsinfrataeniatus* Jan, 1865; **Record Level:** institutionID: ZFMK
- **Type status:** Other material. **Occurrence:** catalogNumber: ZFMK 102509; individualCount: 1; sex: female; **Taxon:** scientificName: *Helicopsinfrataeniatus* Jan, 1865; **Record Level:** institutionID: ZFMK
- **Type status:** Other material. **Occurrence:** catalogNumber: ZFMK 102630; individualCount: 1; sex: female; **Taxon:** scientificName: *Helicopsinfrataeniatus* Jan, 1865; **Location:** country: Brazil; stateProvince: Rio Grande do Sul; locality: Campo Bom (wahrscheinlich); **Record Level:** institutionID: ZFMK
- **Type status:** Other material. **Occurrence:** catalogNumber: ZMB 16436; recordedBy: Mücke leg.; individualCount: 1; sex: female; **Taxon:** scientificName: *Helicopsinfrataeniatus* Jan, 1865; **Location:** country: Brazil; **Record Level:** institutionID: ZMB
- **Type status:** Other material. **Occurrence:** catalogNumber: ZMB 16437; recordedBy: Mücke leg.; individualCount: 1; sex: male; **Taxon:** scientificName: *Helicopsinfrataeniatus* Jan, 1865; **Location:** country: Brazil; **Record Level:** institutionID: ZMB
- **Type status:** Other material. **Occurrence:** catalogNumber: ZMB 16438; recordedBy: Mücke leg.; individualCount: 1; sex: female; **Taxon:** scientificName: *Helicopsinfrataeniatus* Jan, 1865; **Location:** country: Brazil; **Record Level:** institutionID: ZMB
- **Type status:** Other material. **Occurrence:** catalogNumber: ZMB 20606A; recordedBy: R. Hauthal leg.; individualCount: 1; sex: male; **Taxon:** scientificName: *Helicopsinfrataeniatus* Jan, 1865; **Location:** country: Argentina; **Record Level:** institutionID: ZMB
- **Type status:** Other material. **Occurrence:** catalogNumber: ZMB 20606B; recordedBy: R. Hauthal leg.; individualCount: 1; sex: female; **Taxon:** scientificName: *Helicopsinfrataeniatus* Jan, 1865; **Location:** country: Argentina; **Record Level:** institutionID: ZMB
- **Type status:** Other material. **Occurrence:** catalogNumber: ZMB 20606C; recordedBy: R. Hauthal leg.; individualCount: 1; sex: female; **Taxon:** scientificName: *Helicopsinfrataeniatus* Jan, 1865; **Location:** country: Argentina; **Record Level:** institutionID: ZMB
- **Type status:** Other material. **Occurrence:** catalogNumber: ZMB 20607; recordedBy: R. Hauthal leg.; individualCount: 1; sex: male; **Taxon:** scientificName: *Helicopsinfrataeniatus* Jan, 1865; **Location:** country: Argentina; **Record Level:** institutionID: ZMB
- **Type status:** Other material. **Occurrence:** catalogNumber: ZMB 20608A; recordedBy: R. Hauthal leg.; individualCount: 1; sex: male; **Taxon:** scientificName: *Helicopsinfrataeniatus* Jan, 1865; **Location:** country: Argentina; **Record Level:** institutionID: ZMB
- **Type status:** Other material. **Occurrence:** catalogNumber: ZMB 20608B; recordedBy: R. Hauthal leg.; individualCount: 1; sex: male; **Taxon:** scientificName: *Helicopsinfrataeniatus* Jan, 1865; **Location:** country: Argentina; **Record Level:** institutionID: ZMB
- **Type status:** Other material. **Occurrence:** catalogNumber: ZMB 20608C; recordedBy: R. Hauthal leg.; individualCount: 1; sex: female; **Taxon:** scientificName: *Helicopsinfrataeniatus* Jan, 1865; **Location:** country: Argentina; **Record Level:** institutionID: ZMB
- **Type status:** Other material. **Occurrence:** catalogNumber: ZMB 20609A; recordedBy: R. Hauthal leg.; individualCount: 1; sex: male; **Taxon:** scientificName: *Helicopsinfrataeniatus* Jan, 1865; **Location:** country: Argentina; **Record Level:** institutionID: ZMB
- **Type status:** Other material. **Occurrence:** catalogNumber: ZMB 20609B; recordedBy: R. Hauthal leg.; individualCount: 1; sex: female; **Taxon:** scientificName: *Helicopsinfrataeniatus* Jan, 1865; **Location:** country: Argentina; **Record Level:** institutionID: ZMB
- **Type status:** Other material. **Occurrence:** catalogNumber: ZMB 20609C; recordedBy: R. Hauthal leg.; individualCount: 1; sex: male; **Taxon:** scientificName: *Helicopsinfrataeniatus* Jan, 1865; **Location:** country: Argentina; **Record Level:** institutionID: ZMB
- **Type status:** Other material. **Occurrence:** catalogNumber: ZMB 20610A; individualCount: 1; sex: female; **Taxon:** scientificName: *Helicopsinfrataeniatus* Jan, 1865; **Record Level:** institutionID: ZMB
- **Type status:** Other material. **Occurrence:** catalogNumber: ZMB 20610B; individualCount: 1; sex: female; **Taxon:** scientificName: *Helicopsinfrataeniatus* Jan, 1865; **Record Level:** institutionID: ZMB
- **Type status:** Other material. **Occurrence:** catalogNumber: ZMB 20613A; recordedBy: R. Hauthal leg.; individualCount: 1; sex: male; **Taxon:** scientificName: *Helicopsinfrataeniatus* Jan, 1865; **Location:** country: Argentina; **Record Level:** institutionID: ZMB
- **Type status:** Other material. **Occurrence:** catalogNumber: ZMB 20613B; recordedBy: R. Hauthal leg.; individualCount: 1; sex: female; **Taxon:** scientificName: *Helicopsinfrataeniatus* Jan, 1865; **Location:** country: Argentina; **Record Level:** institutionID: ZMB
- **Type status:** Other material. **Occurrence:** catalogNumber: ZMB 20613C; recordedBy: R. Hauthal leg.; individualCount: 1; sex: female; **Taxon:** scientificName: *Helicopsinfrataeniatus* Jan, 1865; **Location:** country: Argentina; **Record Level:** institutionID: ZMB
- **Type status:** Other material. **Occurrence:** catalogNumber: ZMB 20613D; recordedBy: R. Hauthal leg.; individualCount: 1; sex: female; **Taxon:** scientificName: *Helicopsinfrataeniatus* Jan, 1865; **Location:** country: Argentina; **Record Level:** institutionID: ZMB
- **Type status:** Other material. **Occurrence:** catalogNumber: ZMB 20613E; recordedBy: R. Hauthal leg.; individualCount: 1; sex: female; **Taxon:** scientificName: *Helicopsinfrataeniatus* Jan, 1865; **Location:** country: Argentina; **Record Level:** institutionID: ZMB
- **Type status:** Other material. **Occurrence:** catalogNumber: ZMB 20613F; recordedBy: R. Hauthal leg.; individualCount: 1; sex: female; **Taxon:** scientificName: *Helicopsinfrataeniatus* Jan, 1865; **Location:** country: Argentina; **Record Level:** institutionID: ZMB
- **Type status:** Other material. **Occurrence:** catalogNumber: ZMB 6373; recordedBy: R. Hensel leg.; individualCount: 1; sex: male; **Taxon:** scientificName: *Helicopsinfrataeniatus* Jan, 1865; **Location:** country: Brazil; stateProvince: Rio Grande do Sul; locality: Porto Alegre; **Record Level:** institutionID: ZMB
- **Type status:** Other material. **Occurrence:** catalogNumber: ZMB 6840A; recordedBy: R. Hensel leg.; individualCount: 1; sex: female; **Taxon:** scientificName: *Helicopsinfrataeniatus* Jan, 1865; **Location:** country: Brazil; stateProvince: Rio Grande do Sul; locality: Porto Alegre; **Record Level:** institutionID: ZMB
- **Type status:** Other material. **Occurrence:** catalogNumber: ZMB 6840B; recordedBy: R. Hensel leg.; individualCount: 1; sex: female; **Taxon:** scientificName: *Helicopsinfrataeniatus* Jan, 1865; **Location:** country: Brazil; stateProvince: Rio Grande do Sul; locality: Porto Alegre; **Record Level:** institutionID: ZMB
- **Type status:** Other material. **Occurrence:** catalogNumber: ZMB 79245; individualCount: 1; sex: female; **Taxon:** scientificName: *Helicopsinfrataeniatus* Jan, 1865; **Record Level:** institutionID: ZMB
- **Type status:** Other material. **Occurrence:** catalogNumber: ZMB 79246; individualCount: 1; sex: female; **Taxon:** scientificName: *Helicopsinfrataeniatus* Jan, 1865; **Record Level:** institutionID: ZMB
- **Type status:** Other material. **Occurrence:** catalogNumber: ZMB 79247; individualCount: 1; sex: male; **Taxon:** scientificName: *Helicopsinfrataeniatus* Jan, 1865; **Record Level:** institutionID: ZMB
- **Type status:** Other material. **Occurrence:** catalogNumber: ZMB 79248; individualCount: 1; sex: male; **Taxon:** scientificName: *Helicopsinfrataeniatus* Jan, 1865; **Record Level:** institutionID: ZMB
- **Type status:** Other material. **Occurrence:** catalogNumber: ZMB 79249; recordedBy: R. Hensel leg.; individualCount: 1; sex: male; **Taxon:** scientificName: *Helicopsinfrataeniatus* Jan, 1865; **Location:** country: Brazil; stateProvince: Rio Grande do Sul; locality: Porto Alegre; **Record Level:** institutionID: ZMB
- **Type status:** Other material. **Occurrence:** catalogNumber: ZMB 79250; recordedBy: R. Hensel leg.; individualCount: 1; sex: male; **Taxon:** scientificName: *Helicopsinfrataeniatus* Jan, 1865; **Location:** country: Brazil; stateProvince: Rio Grande do Sul; locality: Porto Alegre; **Record Level:** institutionID: ZMB
- **Type status:** Other material. **Occurrence:** catalogNumber: ZMB 89646; individualCount: 1; sex: male; **Taxon:** scientificName: *Helicopsinfrataeniatus* Jan, 1865; **Record Level:** institutionID: ZMB
- **Type status:** Other material. **Occurrence:** catalogNumber: ZMB 89647; individualCount: 1; sex: male; **Taxon:** scientificName: *Helicopsinfrataeniatus* Jan, 1865; **Record Level:** institutionID: ZMB

#### Diagnosis

This species can be distinguished from all its congeners, except *H.carinicaudus*, *H.nentur* and *H.tapajonicus* by the combination of a uniform or longitudinally striped dorsum, a cream or red venter with 1–3 dark stripes or darkly checkered and 17–20 dorsal scale rows at mid-body (versus 20–23 in *H.trivittatus*). From *H.nentur*, it differs in having a semi-divided nasal scale, whereas *H.nentur* has an entire nasal scale. From *H.carinicaudus*, it differs in having a cream or red venter with 1–3 dark stripes or darkly checkered or black with light spots (versus two series of dark semi-lunar marks in *H.carinicaudus*). In some specimens, intermediate patterns are observed. From *H.tapajonicus*, it differs in having strongly keeled dorsal scales, whereas *H.tapajonicus* has only a weak dorsal keeling. Additionally, *H.tapajonicus* possesses a ventrolateral greenish stripe, which is absent in *H.infrataeniatus* (for information on references, see Suppl. material 3). Furthermore, *H.tapajonicus* and *H.infrataeniatus* have allopatric distribution ranges.

#### Distribution

*Helicopsinfrataeniatus* is recorded from the southern Brazilian States Mato Grosso do Sul, Sao Paulo, Parana, Santa Catarina and Rio Grande do Sul and from Paraguay, Uruguay and north-western Argentina Carreira Vidal et al. (2005) (Fig. 2b).

#### Morphology remark

Kawashita-Ribeiro et al. (2013) reported subcaudal keels in *H.infrataeniatus*. In contrast, 57 of the 58 examined specimens during this study showed smooth subcaudal scales. Only SMNS 3065 had subcaudal keels (see Tables 1, 2).

### Helicop leopardinus

(Schlegel, 1837)

5896BB55-7D33-5504-A57D-286F2BF8C992

#### Materials

- **Type status:** Other material. **Occurrence:** catalogNumber: MTKD 15506; individualCount: 1; sex: female; **Taxon:** scientificName: *Helicopsleopardinus* (Schlegel, 1837); **Location:** country: Columbia; **Record Level:** institutionID: MTKD
- **Type status:** Other material. **Occurrence:** catalogNumber: MTKD 27443; recordedBy: Strauss-Hiller leg.; individualCount: 1; sex: female; **Taxon:** scientificName: *Helicopsleopardinus* (Schlegel, 1837); **Location:** country: Argentina; stateProvince: Cordoba; locality: San Franciso; **Record Level:** institutionID: MTKD
- **Type status:** Other material. **Occurrence:** catalogNumber: MTKD 28115; recordedBy: Strauss-Hiller leg.; individualCount: 1; sex: female; **Taxon:** scientificName: *Helicopsleopardinus* (Schlegel, 1837); **Location:** country: Argentina; stateProvince: Cordoba; locality: San Franciso; **Record Level:** institutionID: MTKD
- **Type status:** Other material. **Occurrence:** catalogNumber: MTKD 28716; recordedBy: Strauss-Hiller leg.; individualCount: 1; sex: male; **Taxon:** scientificName: *Helicopsleopardinus* (Schlegel, 1837); **Location:** country: Argentina; locality: Central Chaco; **Record Level:** institutionID: MTKD
- **Type status:** Other material. **Occurrence:** catalogNumber: MTKD 29825; recordedBy: Strauss-Hiller leg.; individualCount: 1; sex: female; **Taxon:** scientificName: *Helicopsleopardinus* (Schlegel, 1837); **Location:** country: Argentina; stateProvince: Chaco; locality: Resistencia; **Record Level:** institutionID: MTKD
- **Type status:** Other material. **Occurrence:** catalogNumber: SMF 100015; recordedBy: M. Jansen leg.; individualCount: 1; sex: female; **Taxon:** scientificName: *Helicopsleopardinus* (Schlegel, 1837); **Location:** country: Bolivia; stateProvince: Santa Cruz; locality: Velasco, Campamento; verbatimLocality: 185; verbatimLatitude: -15°10.493; verbatimLongitude: -61°0.968; **Event:** year: 2007; **Record Level:** institutionID: SMF
- **Type status:** Other material. **Occurrence:** catalogNumber: SMF 17807; recordedBy: I. Schumacher leg.; individualCount: 1; sex: female; **Taxon:** scientificName: *Helicopsleopardinus* (Schlegel, 1837); **Location:** country: Brazil; stateProvince: Mato Grosso; locality: Cujapa; **Event:** year: 1885; **Record Level:** institutionID: SMF
- **Type status:** Other material. **Occurrence:** catalogNumber: SMF 17809; recordedBy: O. Boettger leg.; individualCount: 1; sex: male; **Taxon:** scientificName: *Helicopsleopardinus* (Schlegel, 1837); **Location:** country: Brazil; locality: Northern Brazil; **Event:** year: 1897; **Record Level:** institutionID: SMF
- **Type status:** Other material. **Occurrence:** catalogNumber: SMF 67860; recordedBy: M. Schetty leg.; individualCount: 1; sex: female; **Taxon:** scientificName: *Helicopsleopardinus* (Schlegel, 1837); **Location:** country: Paraguay; **Event:** year: 1972; **Record Level:** institutionID: SMF
- **Type status:** Other material. **Occurrence:** catalogNumber: SMF 67861; recordedBy: M. Schetty leg.; individualCount: 1; sex: female; **Taxon:** scientificName: *Helicopsleopardinus* (Schlegel, 1837); **Location:** country: Paraguay; **Event:** year: 1972; **Record Level:** institutionID: SMF
- **Type status:** Other material. **Occurrence:** catalogNumber: SMF 67862; recordedBy: W.v.d. Wall leg.; individualCount: 1; sex: female; **Taxon:** scientificName: *Helicopsleopardinus* (Schlegel, 1837); **Location:** country: Paraguay; **Event:** year: 1972; **Record Level:** institutionID: SMF
- **Type status:** Other material. **Occurrence:** catalogNumber: ZFMK 36339; individualCount: 1; sex: male; **Taxon:** scientificName: *Helicopsleopardinus* (Schlegel, 1837); **Location:** country: Brazil; **Record Level:** institutionID: ZFMK
- **Type status:** Other material. **Occurrence:** catalogNumber: ZFMK 59774; individualCount: 1; sex: female; **Taxon:** scientificName: *Helicopsleopardinus* (Schlegel, 1837); **Location:** country: Paraguay; stateProvince: Asunción; locality: Pilcomayo; **Record Level:** institutionID: ZFMK
- **Type status:** Other material. **Occurrence:** catalogNumber: ZFMK 59775; individualCount: 1; sex: male; **Taxon:** scientificName: *Helicopsleopardinus* (Schlegel, 1837); **Location:** country: Paraguay; stateProvince: Asunción; locality: Pilcomayo; **Record Level:** institutionID: ZFMK
- **Type status:** Other material. **Occurrence:** catalogNumber: ZFMK 60153; individualCount: 1; sex: male; **Taxon:** scientificName: *Helicopsleopardinus* (Schlegel, 1837); **Location:** country: Bolivia; stateProvince: Santa Cruz; locality: between Florida & Meura, Rio Paraguay; **Record Level:** institutionID: ZFMK
- **Type status:** Other material. **Occurrence:** catalogNumber: ZFMK 62836; individualCount: 1; sex: female; **Taxon:** scientificName: *Helicopsleopardinus* (Schlegel, 1837); **Location:** country: Bolivia; stateProvince: Beni; locality: Campamento Encanto; **Record Level:** institutionID: ZFMK
- **Type status:** Other material. **Occurrence:** catalogNumber: ZMB 10749; recordedBy: R. Rohde leg.; individualCount: 1; sex: male; **Taxon:** scientificName: *Helicopsleopardinus* (Schlegel, 1837); **Location:** country: Paraguay; **Record Level:** institutionID: ZFMK
- **Type status:** Other material. **Occurrence:** catalogNumber: ZMB 20611A; recordedBy: R. Hauthal leg.; individualCount: 1; sex: female; **Taxon:** scientificName: *Helicopsleopardinus* (Schlegel, 1837); **Location:** country: Argentina; **Record Level:** institutionID: ZMB
- **Type status:** Other material. **Occurrence:** catalogNumber: ZMB 20611B; recordedBy: R. Hauthal leg.; individualCount: 1; sex: female; **Taxon:** scientificName: *Helicopsleopardinus* (Schlegel, 1837); **Location:** country: Argentina; **Record Level:** institutionID: ZMB
- **Type status:** Other material. **Occurrence:** catalogNumber: ZMB 26040A; recordedBy: Neumayer leg.; individualCount: 1; sex: male; **Taxon:** scientificName: *Helicopsleopardinus* (Schlegel, 1837); **Location:** country: Argentina; stateProvince: Salta; locality: Tartagal; **Record Level:** institutionID: ZMB
- **Type status:** Other material. **Occurrence:** catalogNumber: ZMB 26040B; recordedBy: Neumayer leg.; individualCount: 1; **Taxon:** scientificName: *Helicopsleopardinus* (Schlegel, 1837); **Location:** country: Argentina; stateProvince: Salta; locality: Tartagal; **Record Level:** institutionID: ZMB
- **Type status:** Other material. **Occurrence:** catalogNumber: ZMB 7545; recordedBy: O. Wucherer leg.; individualCount: 1; sex: male; **Taxon:** scientificName: *Helicopsleopardinus* (Schlegel, 1837); **Location:** country: Brazil; stateProvince: Bahia; **Record Level:** institutionID: ZMB
- **Type status:** Other material. **Occurrence:** catalogNumber: ZMB 89665; individualCount: 1; sex: female; **Taxon:** scientificName: *Helicopsleopardinus* (Schlegel, 1837); **Record Level:** institutionID: ZMB
- **Type status:** Other material. **Occurrence:** catalogNumber: ZSM 1026/2010; recordedBy: G. Walter leg.; individualCount: 1; sex: female; **Taxon:** scientificName: *Helicopsleopardinus* (Schlegel, 1837); **Location:** country: Paraguay; stateProvince: Alto Paraguguay; locality: Puerto Sastre; **Event:** year: 1931; **Record Level:** institutionID: ZMB
- **Type status:** Other material. **Occurrence:** catalogNumber: ZSM 1027/2010; recordedBy: G. Walter leg.; individualCount: 1; sex: male; **Taxon:** scientificName: *Helicopsleopardinus* (Schlegel, 1837); **Location:** country: Paraguay; stateProvince: Alto Paraguguay; locality: Puerto Sastre; **Event:** year: 1931; **Record Level:** institutionID: ZSM
- **Type status:** Other material. **Occurrence:** catalogNumber: ZSM 1028/2010; recordedBy: G. Walter leg.; individualCount: 1; sex: male; **Taxon:** scientificName: *Helicopsleopardinus* (Schlegel, 1837); **Location:** country: Paraguay; stateProvince: Alto Paraguguay; locality: Puerto Sastre; **Event:** year: 1931; **Record Level:** institutionID: ZSM
- **Type status:** Other material. **Occurrence:** catalogNumber: ZSM 1029/2010; recordedBy: G. Walter leg.; individualCount: 1; sex: female; **Taxon:** scientificName: *Helicopsleopardinus* (Schlegel, 1837); **Location:** country: Paraguay; stateProvince: Alto Paraguguay; locality: Puerto Sastre; **Event:** year: 1931; **Record Level:** institutionID: ZSM
- **Type status:** Other material. **Occurrence:** catalogNumber: ZSM 1030/2010; recordedBy: G. Walter leg.; individualCount: 1; sex: female; **Taxon:** scientificName: *Helicopsleopardinus* (Schlegel, 1837); **Location:** country: Paraguay; stateProvince: Alto Paraguguay; locality: Puerto Sastre; **Event:** year: 1931; **Record Level:** institutionID: ZSM
- **Type status:** Other material. **Occurrence:** catalogNumber: ZSM 1031/2010; recordedBy: G. Walter leg.; individualCount: 1; sex: female; **Taxon:** scientificName: *Helicopsleopardinus* (Schlegel, 1837); **Location:** country: Paraguay; stateProvince: Alto Paraguguay; locality: Puerto Sastre; **Event:** year: 1931; **Record Level:** institutionID: ZSM
- **Type status:** Other material. **Occurrence:** catalogNumber: ZSM 1032/2010; recordedBy: G. Walter leg.; individualCount: 1; sex: female; **Taxon:** scientificName: *Helicopsleopardinus* (Schlegel, 1837); **Location:** country: Paraguay; stateProvince: Alto Paraguguay; locality: Puerto Sastre; **Event:** year: 1931; **Record Level:** institutionID: ZSM
- **Type status:** Other material. **Occurrence:** catalogNumber: ZSM 1033/2010; recordedBy: G. Walter leg.; individualCount: 1; sex: female; **Taxon:** scientificName: *Helicopsleopardinus* (Schlegel, 1837); **Location:** country: Paraguay; stateProvince: Alto Paraguguay; locality: Puerto Sastre; **Event:** year: 1931; **Record Level:** institutionID: ZSM
- **Type status:** Other material. **Occurrence:** catalogNumber: ZSM 1034/2010; recordedBy: G. Walter leg.; individualCount: 1; sex: female; **Taxon:** scientificName: *Helicopsleopardinus* (Schlegel, 1837); **Location:** country: Paraguay; stateProvince: Alto Paraguguay; locality: Puerto Sastre; **Event:** year: 1931; **Record Level:** institutionID: ZSM
- **Type status:** Other material. **Occurrence:** catalogNumber: ZSM 1035/2010; recordedBy: G. Walter leg.; individualCount: 1; sex: female; **Taxon:** scientificName: *Helicopsleopardinus* (Schlegel, 1837); **Location:** country: Paraguay; stateProvince: Alto Paraguguay; locality: Puerto Sastre; **Event:** year: 1931; **Record Level:** institutionID: ZSM
- **Type status:** Other material. **Occurrence:** catalogNumber: ZSM 1036/2010; recordedBy: G. Walter leg.; individualCount: 1; sex: male; **Taxon:** scientificName: *Helicopsleopardinus* (Schlegel, 1837); **Location:** country: Paraguay; stateProvince: Alto Paraguguay; locality: Puerto Sastre; **Event:** year: 1931; **Record Level:** institutionID: ZSM
- **Type status:** Other material. **Occurrence:** catalogNumber: ZSM 1037/2010; recordedBy: G. Walter leg.; individualCount: 1; sex: female; **Taxon:** scientificName: *Helicopsleopardinus* (Schlegel, 1837); **Location:** country: Paraguay; stateProvince: Alto Paraguguay; locality: Puerto Sastre; **Event:** year: 1931; **Record Level:** institutionID: ZSM
- **Type status:** Other material. **Occurrence:** catalogNumber: ZSM 1038/2010; recordedBy: G. Walter leg.; individualCount: 1; sex: female; **Taxon:** scientificName: *Helicopsleopardinus* (Schlegel, 1837); **Location:** country: Paraguay; stateProvince: Alto Paraguguay; locality: Puerto Sastre; **Event:** year: 1931; **Record Level:** institutionID: ZSM
- **Type status:** Other material. **Occurrence:** catalogNumber: ZSM 1039/2010; recordedBy: G. Walter leg.; individualCount: 1; sex: female; **Taxon:** scientificName: *Helicopsleopardinus* (Schlegel, 1837); **Location:** country: Paraguay; stateProvince: Alto Paraguguay; locality: Puerto Sastre; **Event:** year: 1931; **Record Level:** institutionID: ZSM
- **Type status:** Other material. **Occurrence:** catalogNumber: ZSM 1040/2010; recordedBy: G. Walter leg.; individualCount: 1; sex: female; **Taxon:** scientificName: *Helicopsleopardinus* (Schlegel, 1837); **Location:** country: Paraguay; stateProvince: Alto Paraguguay; locality: Puerto Sastre; **Event:** year: 1931; **Record Level:** institutionID: ZSM
- **Type status:** Other material. **Occurrence:** catalogNumber: ZSM 134/1947; recordedBy: Zimmermann leg.; individualCount: 1; sex: female; **Taxon:** scientificName: *Helicopsleopardinus* (Schlegel, 1837); **Location:** country: Bolivia; stateProvince: Departamento Beni; locality: Rio Madre de Dios; **Event:** year: <1923?; **Record Level:** institutionID: ZSM
- **Type status:** Other material. **Occurrence:** catalogNumber: ZSM 1523/0; recordedBy: Spix & Martius leg.; individualCount: 1; sex: male; **Taxon:** scientificName: *Helicopsleopardinus* (Schlegel, 1837); **Location:** country: Brazil; **Event:** year: 1817-1820; **Record Level:** institutionID: ZSM
- **Type status:** Other material. **Occurrence:** catalogNumber: ZSM 172/1929; recordedBy: I. Deutsche Chaco-Expedition leg.; individualCount: 1; sex: female; **Taxon:** scientificName: *Helicopsleopardinus* (Schlegel, 1837); **Location:** country: Bolivia; stateProvince: Santa Cruz; locality: San Fermin (100 km nördlich Puerto Suarez); **Event:** year: 1926; **Record Level:** institutionID: ZSM
- **Type status:** Other material. **Occurrence:** catalogNumber: ZSM 268/2017; recordedBy: K. König leg.; individualCount: 1; sex: female; **Taxon:** scientificName: *Helicopsleopardinus* (Schlegel, 1837); **Location:** country: Argentina; stateProvince: Santa Fe; locality: Rosario de Santa Fe; **Event:** year: 1903-1906; **Record Level:** institutionID: ZSM
- **Type status:** Other material. **Occurrence:** catalogNumber: ZSM 269/2017; recordedBy: von Stromer leg.; individualCount: 1; sex: female; **Taxon:** scientificName: *Helicopsleopardinus* (Schlegel, 1837); **Location:** country: Argentina; stateProvince: Santa Fe; locality: Rosario de Santa Fe; **Event:** year: 1907; **Record Level:** institutionID: ZSM
- **Type status:** Other material. **Occurrence:** catalogNumber: ZSM 270/2017; recordedBy: von Stromer leg.; individualCount: 1; sex: female; **Taxon:** scientificName: *Helicopsleopardinus* (Schlegel, 1837); **Location:** country: Argentina; stateProvince: Santa Fe; locality: Rosario de Santa Fe; **Event:** year: 1907; **Record Level:** institutionID: ZSM

#### Diagnosis

*Helicopsleopardinus* is distinguished from all its congeners, except *H.danieli* and *H.gomesi* by the combination of a greyish-olive to greyish-brown dorsum with 4–5 series of alternating dark spots, absent intergenials and 18–22 dorsal scale rows at mid-body (versus 23–26 in *H.polylepis*). From *H.danieli*, it differs in having a cream, yellow or red venter, checkered or banded black or both (versus cream venter with two medial rows of black semi-lunar marks). From *H.gomesi*, it differs in having no subcaudal keels; (for information on references, see Suppl. material 3).

#### Distribution

*Helicopsleopardinus* records range from north-western Argentina to the Estuary of the Amazon and from Ecuador to the Brazilian State Bahia. There are nearly no records in the south-eastern provinces of Brazil (Fig. 2c).

#### Morphology remark

ZSM 134/1947, a female, possesses 109 subcaudals (versus 53–88 in females of *H.leopardinus*). We interpret this as an abnormality (see also Tables 1, 2).

### Helicops modestus

Günther, 1861

B8BD04A5-6D12-5ABD-9ACD-B143EF09C5C8

#### Materials

- **Type status:** Other material. **Occurrence:** catalogNumber: SMF 17802; recordedBy: I. Duschanek leg.; individualCount: 1; sex: female; **Taxon:** scientificName: *Helicopsmodestus* Günther, 1861; **Location:** country: Brazil; stateProvince: Sao Paulo; **Event:** year: 1881; **Record Level:** institutionID: SMF
- **Type status:** Other material. **Occurrence:** catalogNumber: SMF 17803; recordedBy: I. Duschanek leg.; individualCount: 1; sex: female; **Taxon:** scientificName: *Helicopsmodestus* Günther, 1861; **Location:** country: Brazil; stateProvince: Sao Paulo; **Event:** year: 1881; **Record Level:** institutionID: SMF
- **Type status:** Other material. **Occurrence:** catalogNumber: SMF 17804; recordedBy: C. Müller leg.; individualCount: 1; sex: female; **Taxon:** scientificName: *Helicopsmodestus* Günther, 1861; **Location:** country: Brazil; stateProvince: Sao Paulo; **Event:** year: 1876; **Record Level:** institutionID: SMF
- **Type status:** Other material. **Occurrence:** catalogNumber: SMF 17805; recordedBy: C. Müller leg.; individualCount: 1; sex: female; **Taxon:** scientificName: *Helicopsmodestus* Günther, 1861; **Location:** country: Brazil; stateProvince: Sao Paulo; **Event:** year: 1876; **Record Level:** institutionID: SMF
- **Type status:** Other material. **Occurrence:** catalogNumber: SMF 17806; individualCount: 1; sex: male; **Taxon:** scientificName: *Helicopsmodestus* Günther, 1861; **Location:** country: Brazil; stateProvince: Sao Paulo; **Record Level:** institutionID: SMF
- **Type status:** Other material. **Occurrence:** catalogNumber: SMF 49724; recordedBy: M. Schetty leg.; individualCount: 1; sex: female; **Taxon:** scientificName: *Helicopsmodestus* Günther, 1861; **Location:** country: Brazil; stateProvince: Sao Paulo; locality: Umgebung von Sao Paulo; **Event:** year: 1955; **Record Level:** institutionID: SMF
- **Type status:** Other material. **Occurrence:** catalogNumber: SMF 49725; recordedBy: M. Schetty leg.; individualCount: 1; sex: female; **Taxon:** scientificName: *Helicopsmodestus* Günther, 1861; **Location:** country: Brazil; stateProvince: Sao Paulo; locality: Umgebung von Sao Paulo; **Event:** year: 1955; **Record Level:** institutionID: SMF
- **Type status:** Other material. **Occurrence:** catalogNumber: SMF 49726; recordedBy: M. Schetty leg.; individualCount: 1; sex: female; **Taxon:** scientificName: *Helicopsmodestus* Günther, 1861; **Location:** country: Brazil; stateProvince: Sao Paulo; locality: Umgebung von Sao Paulo; **Event:** year: 1955; **Record Level:** institutionID: SMF
- **Type status:** Other material. **Occurrence:** catalogNumber: SMF 49727; recordedBy: M. Schetty leg.; individualCount: 1; sex: female; **Taxon:** scientificName: *Helicopsmodestus* Günther, 1861; **Location:** country: Brazil; stateProvince: Sao Paulo; locality: Umgebung von Sao Paulo; **Event:** year: 1955; **Record Level:** institutionID: SMF
- **Type status:** Other material. **Occurrence:** catalogNumber: SMF 51207; recordedBy: R. Mertens leg.; individualCount: 1; sex: female; **Taxon:** scientificName: *Helicopsmodestus* Günther, 1861; **Location:** country: Brazil; stateProvince: Sao Paulo; locality: Sao Miguel Paulista; **Event:** year: 1955; **Record Level:** institutionID: SMF
- **Type status:** Other material. **Occurrence:** catalogNumber: SMF 91211; recordedBy: F. Weidinger leg.; individualCount: 1; sex: female; **Taxon:** scientificName: *Helicopsmodestus* Günther, 1861; **Location:** country: Brazil; stateProvince: Embu; locality: 25 km von Sao Paulo; **Event:** year: 1954; **Record Level:** institutionID: SMF
- **Type status:** Other material. **Occurrence:** catalogNumber: ZMB 2266; recordedBy: Reinhardt leg.; individualCount: 1; sex: male; **Taxon:** scientificName: *Helicopsmodestus* Günther, 1861; **Location:** country: Brazil; **Record Level:** institutionID: ZMB

#### Diagnosis

*Helicopsmodestus* differs from all its congeners, except *H.carinicaudus*, *H.danieli*, *H.infrataeniatus*, *H.leopardinus*, *H.phantasma* and *H.tapajonicus* by the absence of subcaudal keels and having 19 dorsal scale rows at anterior body. From the remaining species, it differs in having a black to dark green dorsum with indistinct longitudinal stripes and a nearly uniform light cream venter, sometimes with faint flecks; (for information on references, see Suppl. material 3).

#### Distribution

*Helicopsmodestus* is occurring from the Brazilian Province Bahia to the Province Paraná and seems to range from the east shore of Brazil to the south of Mato Grosso. There is also one literature record from Volta Grande do Xingu in the Brazilian Province Para, near its estuary into the Amazon (Vaz-Silva et al. 2015, Fig. 2d).

### Helicops nentur

Costa, Santana, Leal, Koroiva, Garcia, 2016

938B7A56-BCA5-5786-BE81-CDFDFE82DC40

#### Diagnosis

*Helicopsnentur* differs from all its congeners by the combination of an entire nasal scale and 17 dorsal scale rows at mid-body (versus 25 in *H.boitata*; 25–28 in *H.yacu)*; (for information on references, see Suppl. material 3).

#### Distribution

*Helicopsnentur* is known only from the eastern part of the Brazilian Province Minas Gerais (Fig. 2e).

### Helicops pastazae

Shreve, 1934

B4D05C38-E6EB-5549-977A-486EA8DE1515

#### Materials

- **Type status:** Other material. **Occurrence:** catalogNumber: ZSM 519/2003; recordedBy: J. Foerster leg.; individualCount: 1; sex: female; **Taxon:** scientificName: *Helicopspastazae* Shreve, 1934; **Location:** country: Ecuador; stateProvince: Napo; locality: Virgilio Davila (Borja), Quijos; **Event:** year: <1952; **Record Level:** institutionID: ZSM

#### Diagnosis

*Helicopspastazae* can be distinguished from all other congeners, except *H.hagmanni* and *H.yacu* by the combination of having subcaudal keels and intergenials present. From *H.hagmanni*, it differs in having 72–117 subcaudal scales (versus 50–59 in *H.hagmanni*). From *H.yacu*, it differs in having a semi-divided nasal scale (entire in *H.yacu)*; (for information on references, see Suppl. material 3).

#### Distribution

*Helicopspastazae* is found in the northern part of Ecuador and the eastern part of Venezuela. There are no reports from Colombia (Fig. 2f).

### Helicops petersi

Rossman, 1976

63D8B581-2042-50AC-B942-1AD4E338EC73

#### Diagnosis

*Helicopspetersi* can be distinguished from all its congeners, except *H.yacu* by the combination of present intergenials, absent subcaudal keels and 135–150 ventrals (versus 110–125 in *H.scalaris*; 114–130 in *H.trivittatus*). From *H.yacu*, it differs in having a semi-divided nasal scale (versus entire in *H.yacu)*; (for information on references, see Suppl. material 3).

#### Distribution

*Helicopspetersi* is known only from a very small area in the Ecuadorian Province Napo (Fig. 3a).

### Helicops phantasma

Moraes-da-Silva, Amaro, Nunes, Rodrigues & Curcio, 2021

43552550-F531-5EA6-901B-B8BD0D3B378D

#### Diagnosis

*Helicopsphantasma* can be distinguished from all other congeners by having a dorsal pattern of dark spots fusing to irregular black bands, absent intergenials and 19/19/17–19 dorsal scale rows with moderate keels (versus 25/25/21 in *H.boitata*; 19–21/18–20/16–19 in *H.danieli*; 19/19/19 in *H.gomesi*; 15–22/18–22/16–19 in *H.leopardinus*; 23–25/23–26/17–21 in *H.polylepis*) and hemipenial morphology (for information on references, see Suppl. material 3).

#### Distribution

The species is only known from the Tocantins-Araguaia River Basin in the Provinces Tocantins, Mato Grosso and Maranhão in northern Brazil (Moraes-da-Silva et al. 2021, Fig. 3b).

### Helicops polylepis

Günther, 1861

25B5EB5A-22AC-5A78-AEC9-2871F34F279F

#### Materials

- **Type status:** Other material. **Occurrence:** catalogNumber: MTKD 15507; individualCount: 1; sex: female; **Taxon:** scientificName: *Helicopspolylepis* Günther, 1861; **Location:** country: Columbia; **Record Level:** institutionID: MTKD
- **Type status:** Other material. **Occurrence:** catalogNumber: MTKD 15508; individualCount: 1; sex: female; **Taxon:** scientificName: *Helicopspolylepis* Günther, 1861; **Location:** country: Columbia; **Record Level:** institutionID: MTKD
- **Type status:** Other material. **Occurrence:** catalogNumber: SMF 17821; recordedBy: F. Werner leg.; individualCount: 1; sex: female; **Taxon:** scientificName: *Helicopspolylepis* Günther, 1861; **Location:** country: Bolivia; stateProvince: Chaco; **Record Level:** institutionID: SMF
- **Type status:** Other material. **Occurrence:** catalogNumber: SMF 17822; recordedBy: C.A. Hahn leg.; individualCount: 1; sex: female; **Taxon:** scientificName: *Helicopspolylepis* Günther, 1861; **Location:** country: Bolivia; **Record Level:** institutionID: SMF
- **Type status:** Other material. **Occurrence:** catalogNumber: ZMB 17428; individualCount: 1; sex: male; **Taxon:** scientificName: *Helicopspolylepis* Günther, 1861; **Location:** country: Brazil; stateProvince: Bahia; **Record Level:** institutionID: ZMB
- **Type status:** Other material. **Occurrence:** catalogNumber: ZMB 26215; recordedBy: Stoecker leg.; individualCount: 1; sex: male; **Taxon:** scientificName: *Helicopspolylepis* Günther, 1861; **Location:** country: Bolivia; stateProvince: La Paz; locality: La Paz; **Record Level:** institutionID: ZMB
- **Type status:** Other material. **Occurrence:** catalogNumber: ZMB 30993; individualCount: 1; **Taxon:** scientificName: *Helicopspolylepis* Günther, 1861; **Record Level:** institutionID: ZMB

#### Diagnosis

*Helicopspolylepis* can be distinguished from all its congeners by the combination of absent intergenials, a semi-divided nasal scale and 23–26 dorsal scale rows at mid-body (versus 17–20 in *H.angulatus*; 21–22 in *H.apiaka*; 17–19 in *H.carinidaudus*; 17–20 in *H.infrataeniatus* and *H.modestus*; 18–20 in *H.danieli*; 19 in *H.gomesi*; 18–22 in *H.leopardinus*; 19 in *H.phantasma*; 23–26 in *H.polylepis*; 19 in *H.tapajonicus*; (for information on references, see Suppl. material 3).

#### Distribution

*Helicopspolylepis* is recorded from southern Bolivia to the Amazon Estuary and from Peru to the east of the Brazilian Province Para. There are also two reports from Colombia (Fig. 3c).

### Helicops scalaris

Jan, 1865

299604E1-56C4-5393-A2F2-1DA3A9432BE1

#### Diagnosis

This species can be distinguished from all its congeners, except *H.trivittatus* by the combination of having intergenials, 110–119 ventrals in males and 113–125 in females (versus ♂117–127♀130–138 in *H.hagmanni*; ♂121–134♀130–145 in *H.pastazae*; ♂135–142♀137–150 in *H.petersi*; ♂124♀129–136 in *H.yacu*) and 67–95 subcaudals (versus 55–59 in *H.hagmanni*). From *H.trivittatus*, it differs in having a blotched dorsum, versus striped in *H.trivittatus* (for information on references, see Suppl. material 3).

#### Distribution

*Helicopsscalaris* is known only from the northern border area between Colombia and Venezuela, western and north of Lake Maracaibo in Venezuela (Fig. 3d).

### Helicops tapajonicus

da Frota, 2005

05610BDB-71EC-5524-8BC2-3AC1147DD863

#### Diagnosis

This species can be distinguished from all its congeners by its unique colour pattern, namely the combination of a uniform moss-green dorsum, laterally with a greenish-yellow stripe and a black and greenish-yellow banded venter (for information on references, see Suppl. material 3).

#### Distribution

*Helicopstapajonicus* is only known from two localities at the River Tapajos close to its confluence with the Amazon in the Brazilian State Para (Fig. 3e).

### Helicops trivittatus

(Gray, 1849)

8E0D2599-F4B3-55EB-B85B-2B35F7177580

#### Materials

- **Type status:** Other material. **Occurrence:** catalogNumber: SMF 17798; recordedBy: Z.G. leg.; individualCount: 1; sex: female; **Taxon:** scientificName: *Helicopstrivittatus* (Gray, 1849); **Event:** year: 1915; **Record Level:** institutionID: SMF
- **Type status:** Other material. **Occurrence:** catalogNumber: SMF 45434; recordedBy: K. Müller leg.; individualCount: 1; sex: male; **Taxon:** scientificName: *Helicopstrivittatus* (Gray, 1849); **Location:** country: Brazil; stateProvince: Para; locality: Amazonas; **Event:** year: 1953; **Record Level:** institutionID: SMF
- **Type status:** Other material. **Occurrence:** catalogNumber: ZSM 272/2017; recordedBy: L. Müller leg.; individualCount: 1; sex: female; **Taxon:** scientificName: *Helicopstrivittatus* (Gray, 1849); **Location:** country: Brazil; stateProvince: Pará; locality: Insel Marajó, Cachoeira am mittleren Arary; **Event:** year: 1910; **Record Level:** institutionID: ZSM
- **Type status:** Other material. **Occurrence:** catalogNumber: ZSM 273/2017; recordedBy: L. Müller leg.; individualCount: 1; sex: female; **Taxon:** scientificName: *Helicopstrivittatus* (Gray, 1849); **Location:** country: Brazil; stateProvince: Pará; locality: Insel Marajó, Cachoeira am mittleren Arary; **Event:** year: 1910; **Record Level:** institutionID: ZSM

#### Diagnosis

This species can be distinguished from all congeners by the unique colour pattern, namely a combination of five narrow light stripes on the dorsum and a light venter with black semi-lunar markings, which extend on to the tail (for information on references, see Suppl. material 3).

#### Distribution

*Helicopstrivittatus* is present from the eastern part of the Brazilian Province Para to approximately its borders with Maranhao, Tocantins and northern Mato Grosso. There are no reports of this species from western Para (*Fig. 3f*).

#### Morphology remark

The presence of intergenials seems to be a reliable identification character in all other species of this genus, whereas in *H.trivittatus*, this character shows considerable variation. Intergenials are sometimes present. In our dataset, there were two specimens with and two without intergenials. There is no obvious biogeographical pattern perceiveable (pers. Comm. Antonio Moraes-da-Silva).

### Helicops yacu

Rossman & Dixon, 1975

C885DF1B-28C3-56E9-A8C8-D8729381DF0A

#### Diagnosis

*Helicopsyacu* can be distinguished from all congeners by the combination of having an entire nasal scale and intergenials present (for information on references, see Suppl. material 3).

#### Distribution

*Helicopsyacu* is known only from north-eastern of the Province Loreto, Peru and one locality in north-western Acre, Brazil (Rossman and Dixon 1975, Nogueira et al. 2019, Fig. 4).

#### Taxonomic remark

In Rossman and Abe (1979), the authors express their doubt that *H.yacu* represents a valid species. They state the possibility that individuals assigned to *H.yacu* might represent a subspecies of *H.pastazae*. However, they state that further specimens are needed for an accurate assessment. No further verification of this hypothesis has been made since then.

### Helicops sp.

B75A2520-D197-5254-93E8-2BF0144E7A4D

#### Materials

- **Type status:** Other material. **Occurrence:** catalogNumber: SMF 34035; recordedBy: E. Bresslau leg.; individualCount: 1; sex: female; **Taxon:** scientificName: *Helicops* sp.; **Location:** country: Brazil; stateProvince: Pernambuco; **Event:** year: 1913-1914; **Record Level:** institutionID: SMF

#### Diagnosis

The female specimen SMF 34035 is distinguished from all other congeners, except *H.angulatus*, *H.infrataeniatus* and *H.modestus* by having 17 dorsal scale rows at mid-body and posterior body and 124 ventrals (compared to 111-117 in *H.nentur*). From the rest, it differs in having a black venter with cream, narrow transversal bands, which are approximately a ventral scale wide, often left and right halves are shifted one ventral scale, forming a pattern resembling a chessboard. *Helicopsangulatus* has a banded venter, *Helicopsinfrataeniatus* has a venter either with three black stripes on a cream background or checkered black and cream, sometimes red, *H.modestus* has a uniform cream venter. Additionally, the specimen can be distinguished from *H.infrataeniatus* by its distribution. It originates from the Brazilian Province Pernambuco, whereas *H.infrataeniatus* occurs no further north than Mato Grosso do Sul in Brazil (Pholidosis of specimen 34035, see Table 3; for information on references, see Suppl. material 3).

#### Distribution

The specimen originates from the Province Pernambuco in Brazil, no exact locality is available.

## Identification Keys

### Identification key to the species of *Helicops* Wagler, 1830

**Table:** 

1	Dorsum uniform or with longitudinal stripes	2
–	Dorsum with blotches, spots or transverse bars	8
2	Dorsum tan to dark brown with five rows of narrow light stripes, ventral cream with two uniform rows of dark brown to black semi-lunar marks	*Helicopstrivittatus*
–	Colouration not as above	3
3	Venter cream or yellow with 2–3 rows of black semi-lunar marks, 9–10 infralabials, 128–141 ventrals in males and 128–148 ventrals in females	*H.carinicaudus*
–	Colouration and pholidosis not as above	4
4	Nasal entire	7
–	Nasal semi-divided	5
5	Ventral body cream, with or without faint brown flecks	*H.modestus*
–	Venter contrastingly checkered or with dark longitudinal stripes	6
6	Dorsum uniform moss green, dorsal weakly keeled	*H.tapajonicus*
–	Venter cream or red with 1–3 dark stripes or darkly checkered or black with light spots or intermediate forms; dorsal strongly keeled; dorsum dark brown with pale brown stripes	*H.infrataeniatus*
7	Dorsal scale rows at mid-body 17, reducing to 15 anterior to cloaca; 56 subcaudals in the single known male, 41–52 in females; dorsum uniform dark olive, dark brown or dark grey	*H.nentur*
–	Dorsal scale rows at mid-body 25, reducing to 21 anterior to cloaca; 68 subcaudals in the single known male, unknown in females; dorsum greenish-copper brown with three longitudinal rows of dark, rectangular spots, venter light greyish-brown with two lateral rows of light orange spots	*H.boitata*
8	Intergenials present	9
–	Intergenials absent	13
9	Nasal entire; 85–96 subcaudals in females, unknown in males; 25–28 dorsal scale rows at mid-body, reducing to 18–20 anterior to cloaca; dorsum light to medium grey brown with 4 alternating rows of relatively small dark spots	*H.yacu*
–	Nasal semi-divided; 55–117 subcaudals in males, 51–97 in females; 19–29 dorsal scale rows at mid-body, reducing to 16–23 anterior to cloaca; colouration variable	10
10	55–67 subcaudals in males, 50–53 in females; dorsum grey brown with alternating light and dark circular blotches; northern South America	*H.hagmanni*
–	83–117 subcaudals in males, 50–97 in females; colouration variable	11
11	110–119 ventrals in males, 113–125 in females; subcaudal keels absent; dorsum greyish-tan with 3–5 rows of irregular dark blotches, the vertebral blotches larger than laterals, all 3 usually fused longitudinally; northern South America	*H.scalaris*
–	121–142 subcaudals in males, 130–150 in females; subcaudal keels present; colouration variable	12
12	Weak subcaudal keels present, 121–134 ventrals in males, 130–145 in females; 93–117 subcaudals in males, 72–97 in females; 23–25 dorsal scale rows at mid-body, reducing to 16–19 anterior to cloaca; ventral colouration cream with a series of dark crossbands or alternating checks, light ventral colour extending on to several dorsal scale rows; northern South America	*H.pastazae*
–	Subcaudal keels absent, 135–142 ventrals in males, 137–150 in females; 85–91 subcaudals in males, 67–73 in females; 21–23 dorsal scale rows at mid-body, reducing to 16 anterior to cloaca; ventral colouration cream with a lateral series of dark checks; eastern Andean foothills of Ecuador	*H.petersi*
13	Subcaudal keels present	14
–	Subcaudal keels absent	16
14	103–123 ventrals in males, 104–125 in females; 17–20 dorsal scale rows at mid-body	*H.angulatus*
–	118–132 ventrals in males, 124–132 in females or, if fewer than 124 ventrals in males, then 21–22 dorsal scale rows at mid-body	15
15	19 dorsal scale rows at mid-body; dorsum with dark blotches; one anterior temporal; 71–86 subcaudals in males, 67–73 in females; 125–132 ventrals in males, 128–132 in females	*H.gomesi*
–	21–24 dorsal scale rows at mid-body; dorsum with dark transverse bands; 2–3 anterior temporals; 79–103 subcaudals in males, 80–84 in females; 118–127 ventrals in males, 124–132 in females; northern Mato Grosso, Brazil	*H.apiaka*
16	Dorsum scale rows at mid-body 23–26, reducing to 17–21 anterior to cloaca; 71–101 subcaudals in males, 71–88 in females; 10–13 infralabials; venter dark with pale spots	*H.polylepis*
–	Dorsum scale rows at mid-body 19–22, reducing to 16–19 anterior to cloaca; 64–89 subcaudals in males, 53–76 in females; 8–11 infralabials; venter checkered or banded black and red or cream with two medial rows of black semi-lunar marks, sometimes fused mid-ventrally	17
17	19 dorsal scale rows at anterior and mid-body and 17–19 dorsal scale rows anterior to cloaca; dorsal scales with moderate keels; dark dorsal spots fusing to transversal bands	*H.phantasma*
–	Number of dorsal scale rows different	18
18	Venter checkered or banded black and red; 108–129 ventrals in males, 108–138 in females	*H.leopardinus*
–	Venter cream with two medial rows of black semi-lunar marks, sometimes fused mid-ventrally; 125–135 ventrals in males, 130–141 in females	*H.danieli*

Dichotomous identification key, based on our own examinations and literature (listed in Suppl. material 3).

## Discussion

### Taxonomic discussion

The last published identification key by Peters and Orejas-Miranda (1970) is by now outdated and many taxonomic changes have since taken place. Therefore, we evaluated the suitability of identification characters proposed in literature. Based on this revision, we created a completely restructured identification key. The number of subcaudal and ventral scales show rather large overlaps, often eliminating them as diagnostic characters, for example, the number of ventrals in *H.angulatus* overlaps with the ranges of 13 of the other species . In addition, the number of dorsal scale rows have to be treated cautiously. There is considerable variation (17–29 DSM) and each species has overlaps with at least one other species, with all species, except *H.boitata*, *H.pastazae*, *H.polylepis* and *H.yacu* having individuals with 19–21 DSM.

Peters and Orejas-Miranda (1970) used only the number of preocular scales as a distinguishing character for *H.trivittatus*. According to these authors, this species has two preoculars. We confirm this observation, but found six other species (*H.angulatus*, *H.carinicaudus*, *H.hagmanni*, *H.infrataeniatus*, *H.leopardinus* and *H.modestus*) having either one or two preoculars, eliminating this character as unique for *H.trivittatus*. Our observations are supported by data from Moraes-da-Silva et al. (2019).

Regarding the head scutellation, we found that only the presence of intergenial scales is a stable diagnostic character s in all species, except *H.trivittatus*. In this species, specimens with and without intergenials occur without a geographical pattern (pers. comm. Antonio Moraes-da-Silva).

Colouration seems to be a rather good character for distinguishing some species (e.g. *H.trivttatus*) and species groups, but this might change with further molecular studies and the possible identification of cryptic species. We could not find a pholidotic character in order to distinguish *H.carinicaudus* from *H.infrataeniatus* with neither head scalation showing clear differences nor the ratio TL/SVL. We found them to differ only in ventral colouration, which agrees with Peters and Orejas-Miranda (1970), who regarded *H.infrataeniatus* as a subspecies of *H.carinicaudus*. *Helicopscarinicaudus* has a yellow or cream venter with two series of black semi-lunar marks with small, irregular black spots between these marks, whereas *H.infrataeniatus* has a red to white venter, with three black stripes, checkered black and light or black with light spots. Obviously, *H.carinicaudus* and *H.infrataeniatus* need a closer examination using molecular methods. Achaval Elena (2001) made a similar statement for *H.infrataeniatus*, during the evaluation of the systematics and distribution of nearly all reptile species of Uruguay. A further hint towards the necessity of revisionary work on these species is the documentation of *Helicops* sp. reported in this publication. As described, it resembles *H.infrataeniatus*, but has a considerably different ventral colouration. Moraes-da-Silva et al. (2019) stated that literature on *Helicops* morphology is sometimes contradictory - we agree.

In Murphy et al. (2020), the authors resurrected the species *Helicopscyclops*, which was previously considered to be a synonym of *H.angulatus*. However, the resurrection is only based on a photograph of a single specimen, which was not examined by the authors themselves. We think the observed differences to *H.angulatus* (short snout, shortened chin shields and a distinct dark band between the eyes) could also be explained by individual variation, especially in a species with such a large distribution range. The authors, furthermore, list a higher number of ventral scales (124 ventrals) as diagnostic character, which is well in the range we observed. Therefore, we do not include this species in our key. We encourage a re-evaluation including genetic data and a proper re-description of the species to clarify its taxonomic status.

The presence versus the absence of subcaudal keels seems to be a stable character in most species. However, we found conflicting reports for this character in *H.infrataeniatus* for which Kawashita-Ribeiro et al. (2013) reported subcaudal keels, whereas Moraes-da-Silva et al. (2019) stated that these are absent in this species. Amongst the 58 specimens of this species, only one (SMNS 3065) possessed subcaudal keels. This finding might be explained by variation throughout the large distribution range of this species. It could be a sign that distinct genetic lineages exist, as we experienced this character extremely stable in the other species.

We think it is possible that some species with supposedly large geographical distributions actually comprise species complexes. For example, Murphy et al. (2020) already showed cryptic diversity in what is currently recognised as *H.angulatus*. We expect future integrative studies including genetic data to reveal cryptic and, therefore, yet undiscovered species.

### Geographical extensions

When comparing distribution data from our examined specimens with literature records, we discovered range extensions for five species. For *H.carinicaudus*, we report one specimen collected in 1935 in Porto Alegre, Rio Grande do Sul, Brazil. This would represent a distribution range extension of over 150 km from the closest literature record by Deiques and Cechin (1991). However, this species is not known to occur so far south (Nogueira et al. 2019). It might only have been shipped from Porto Alegre. For *H.danieli*, we report the first specimen for Brazil. The specimen ZMB 9490 has no precise locality data. The nearest literature report of this species to Brazil is only 20 km away from the Columbian-Brazilian border at Mitu, in the Columbian Province Vaupes (Yuki and Castano 1998). For *H.infrataeniatus*, we report an expansion of the distribution range approximately 50 km further west from the nearest record by Giraudo (2001) by specimen SMF 67327. It was collected in Roque Saenz Pena, Chaco, Argentina and represents the northernmost record in the Argentinian Province of Chaco. For the species *H.leopardinus*, we report an extension of the previously known distribution range to the Argentinian Province Salta with specimen ZMB 26040-A collected at Tartagal. Specimen MTKD 27443 was found near San Francisco, Cordoba, Argentina. This specimen represents the first record of the species in this Province, extending the distribution range around 110 km to the west from the nearest records by Nogueira et al. (2019). We extend the distribution range of *Helicopspastazae* to the Province Napo, Ecuador, based on specimen ZMB 519/2003. It was collected in Virgilio Davila, Quijos, Napo, Ecuador. This is around 90 km east of the nearest literature record at Yachana Reserve, Orellana, Ecuador (Whitworth and Beirne 2011). It is conspicuous that there are specimens from eastern Venezuela and from northern Ecuador, but no specimens or reports from adjacent areas in Columbia. It is likely that *H.pastazae* is present in Columbia, but has not been reported, because of missing research and fieldwork. For *H.polylepis*, we report a range extension of approximately 120 km southwest from the nearest record in Puerto Linares, La Paz, Bolivia by Nogueira et al. (2019). Specimen ZMB 26215 was collected in La Paz, La Paz, Bolivia. Furthermore, we report the first specimen from the Province Chuquisaca in Bolivia. Specimen SMF 17821 was collected in Chaco, Chuquisaca, Bolivia. This extends the distribution range approx. 130 km from the nearest record in Caranavi, La Paz, Bolivia by Nogueira et al. (2019). Finally, we report this species from the Province of Bahia in Brazil. Specimen ZMB 17428 was collected there, but no exact locality data are available.

The number of range extensions we report shows that the distribution ranges of the species in this genus are not yet well known. In order to change that, a comprehensive examination of collected material at an international level and additional fieldwork are required.

## Supplementary Material

XML Treatment for Helicops angulatus

XML Treatment for Helicops apiaka

XML Treatment for Helicops boitata

XML Treatment for Helicops carinicaudus

XML Treatment for Helicops danieli

XML Treatment for Helicops gomesi

XML Treatment for Helicops hagmanni

XML Treatment for Helicops infrataeniatus

XML Treatment for Helicop leopardinus

XML Treatment for Helicops modestus

XML Treatment for Helicops nentur

XML Treatment for Helicops pastazae

XML Treatment for Helicops petersi

XML Treatment for Helicops phantasma

XML Treatment for Helicops polylepis

XML Treatment for Helicops scalaris

XML Treatment for Helicops tapajonicus

XML Treatment for Helicops trivittatus

XML Treatment for Helicops yacu

XML Treatment for Helicops sp.

A2F38E76-B239-5D3F-9D4C-7B733FA255D5 10.3897/BDJ.10.e69234.suppl1 Supplementary material 1 Examination results Data type morphological Brief description Examination results of all 190 specimens examined in this study. File: oo_531784.tsv https://binary.pensoft.net/file/531784 Yannis Schöneberg, Gunther Köhler

752130E9-CFB5-55CB-9467-09298E73407E 10.3897/BDJ.10.e69234.suppl2 Supplementary material 2 References for all locality records extracted from literature Data type occurences Brief description This table contains the description, coordinates and the reference of all used distribution points, which were extracted from literature. File: oo_597917.tsv https://binary.pensoft.net/file/597917 Yannis Schöneberg, Gunther Köhler

724B7361-C3E8-5E7A-BA4B-06690D6C98B6 10.3897/BDJ.10.e69234.suppl3 Supplementary material 3 References for the morphological data Data type morphological Brief description This file contains all references used for the assessment of the morphological traits. File: oo_531786.txt https://binary.pensoft.net/file/531786 Yannis Schöneberg, Gunther Köhler

## Figures and Tables

**Figure 1a.**
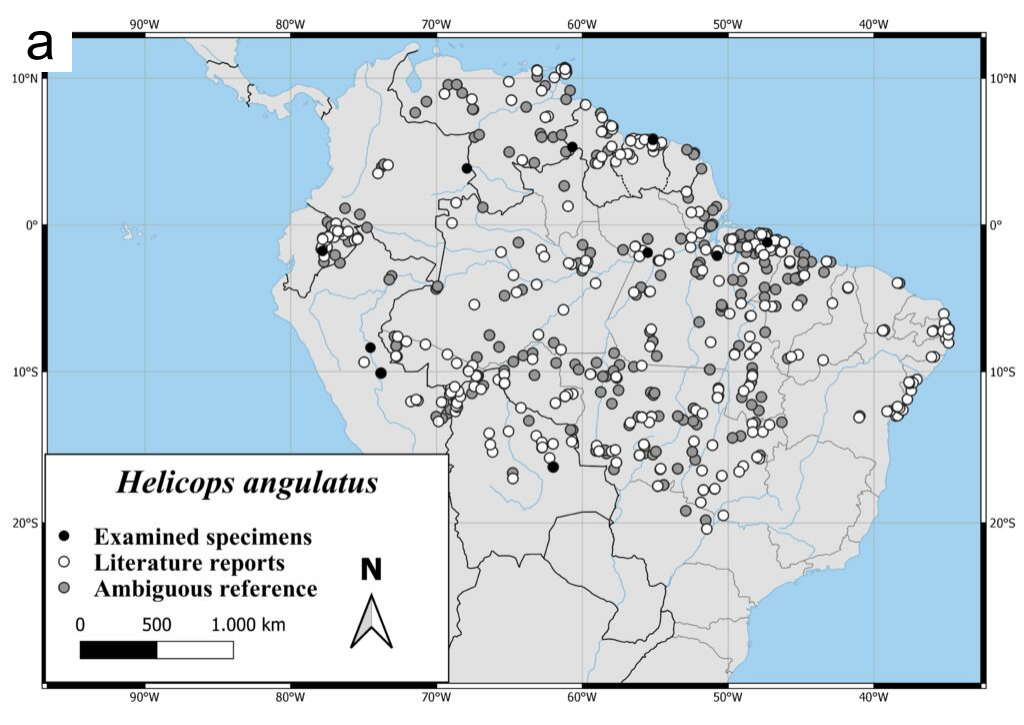


**Figure 1b.**
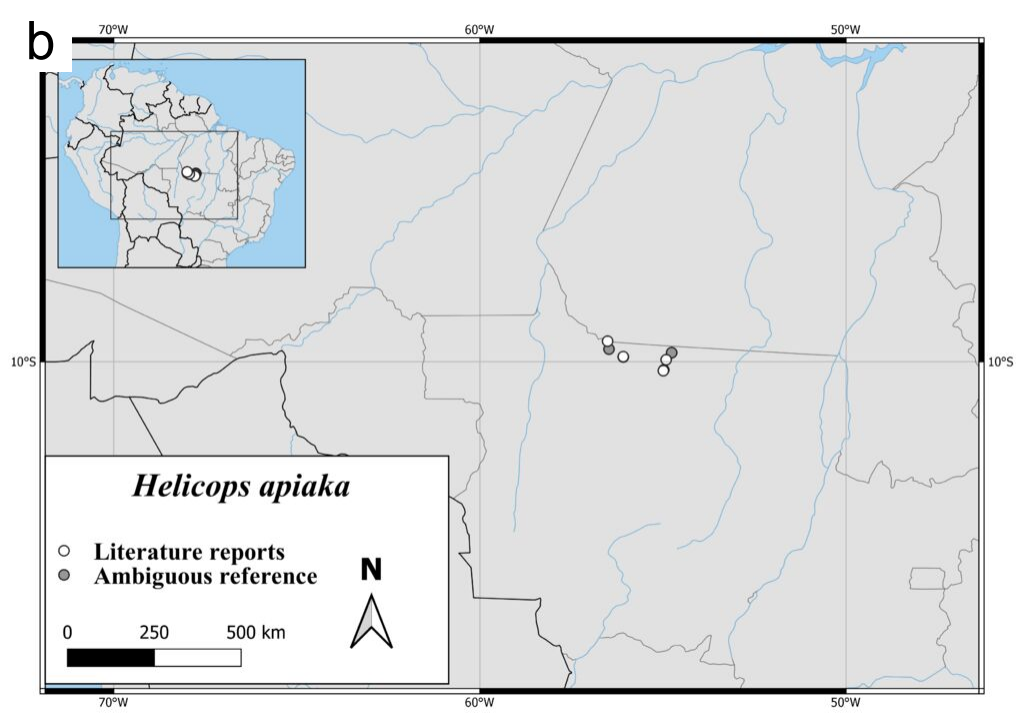


**Figure 1c.**
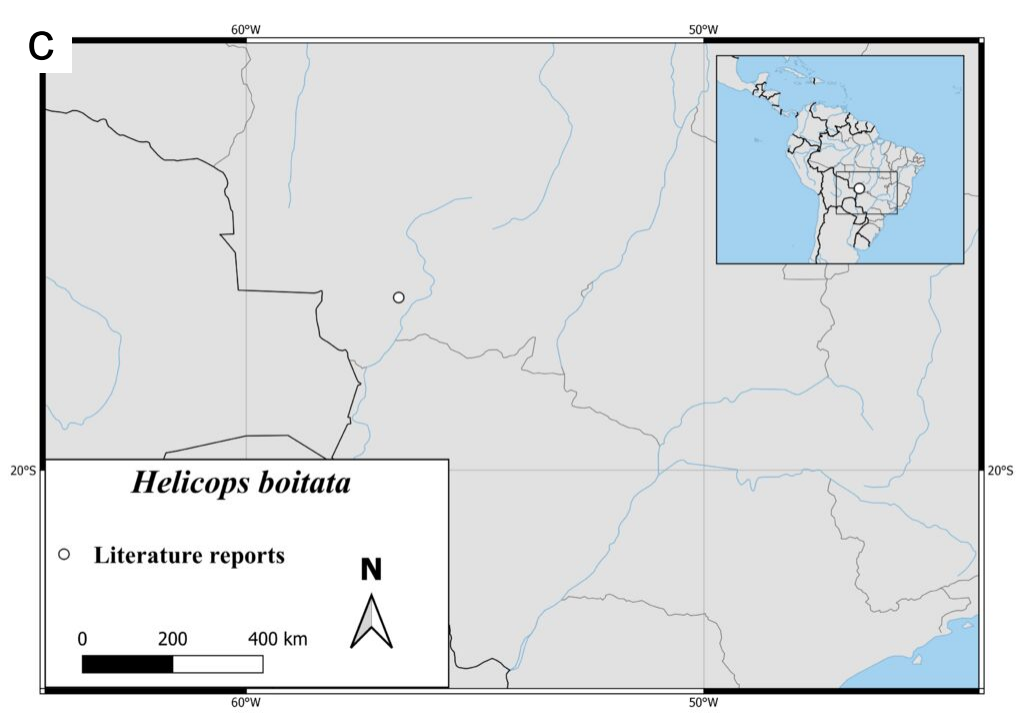


**Figure 1d.**
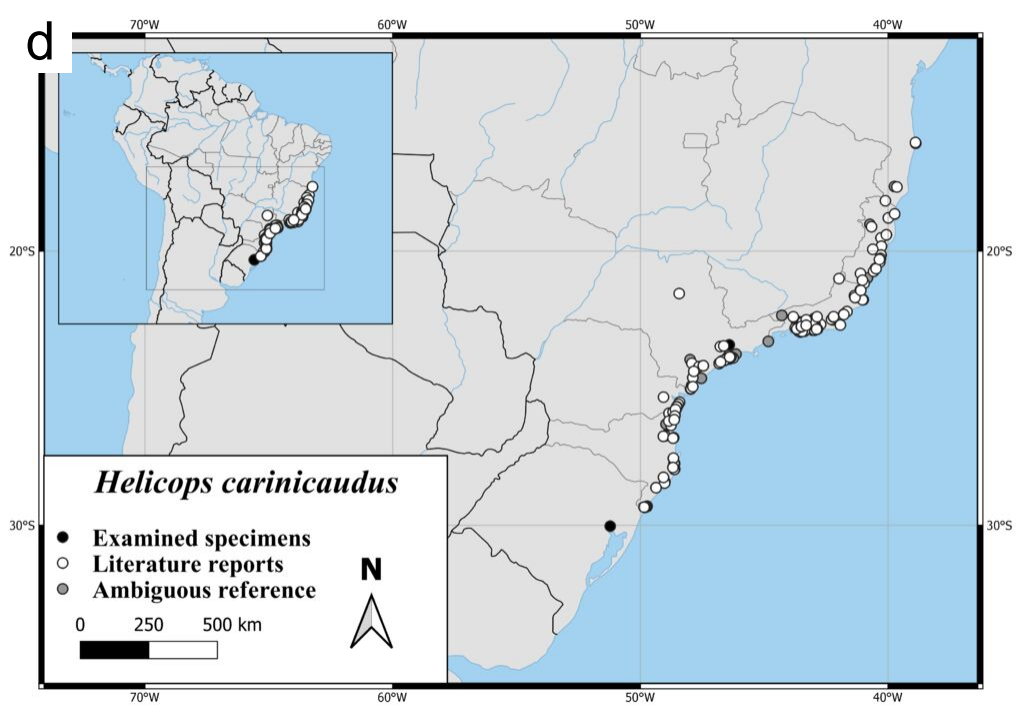


**Figure 1e.**
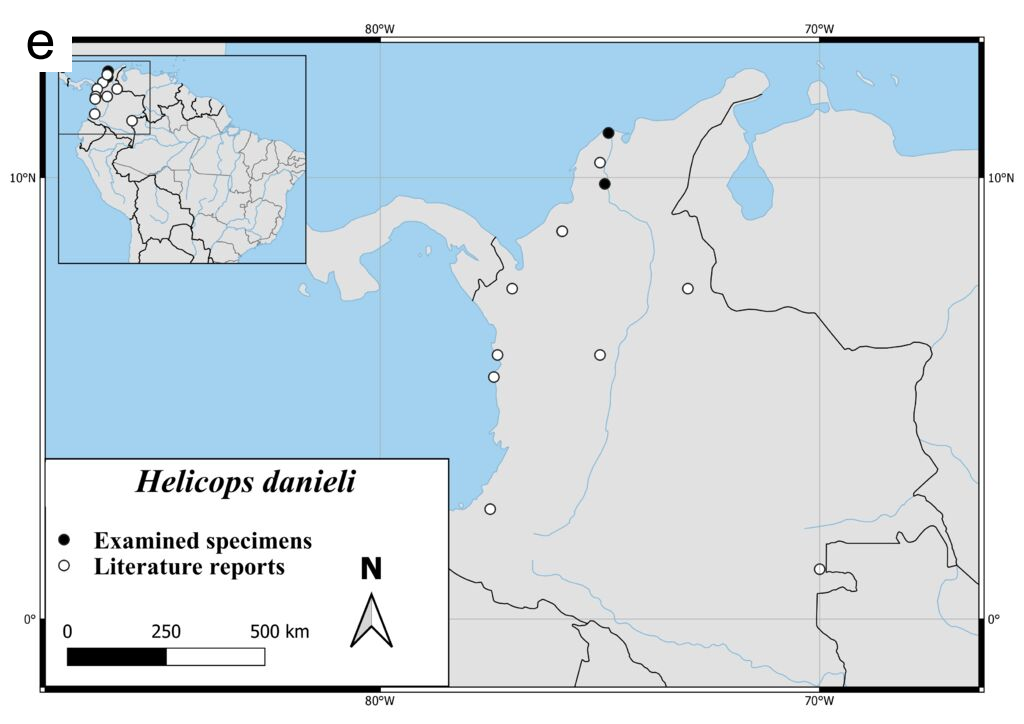


**Figure 1f.**
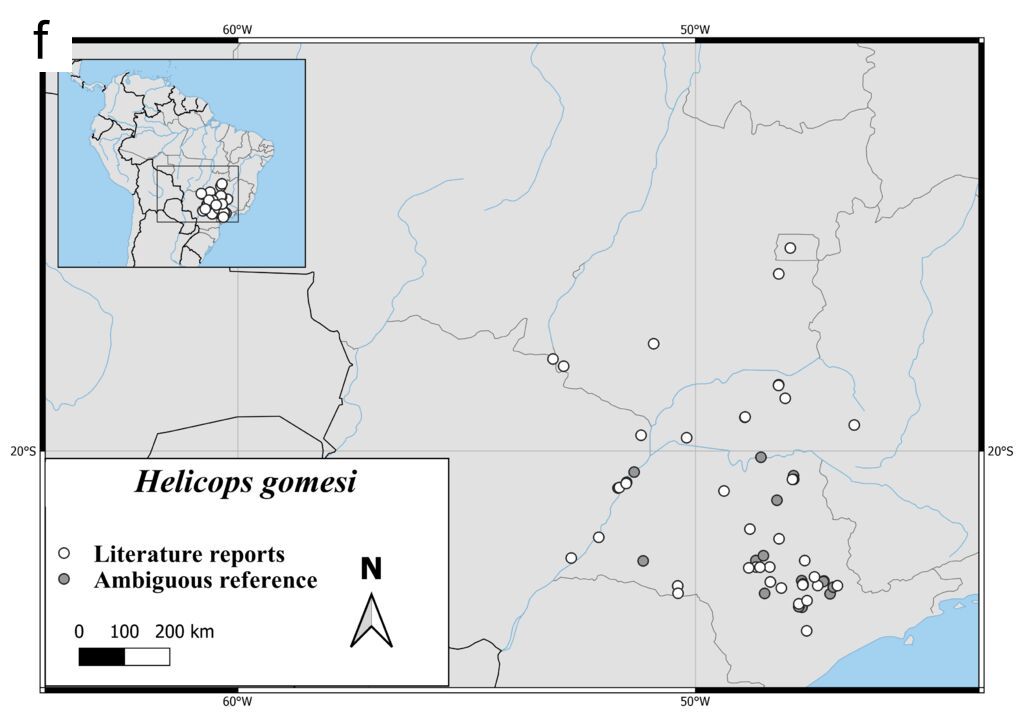


**Figure 2a.**
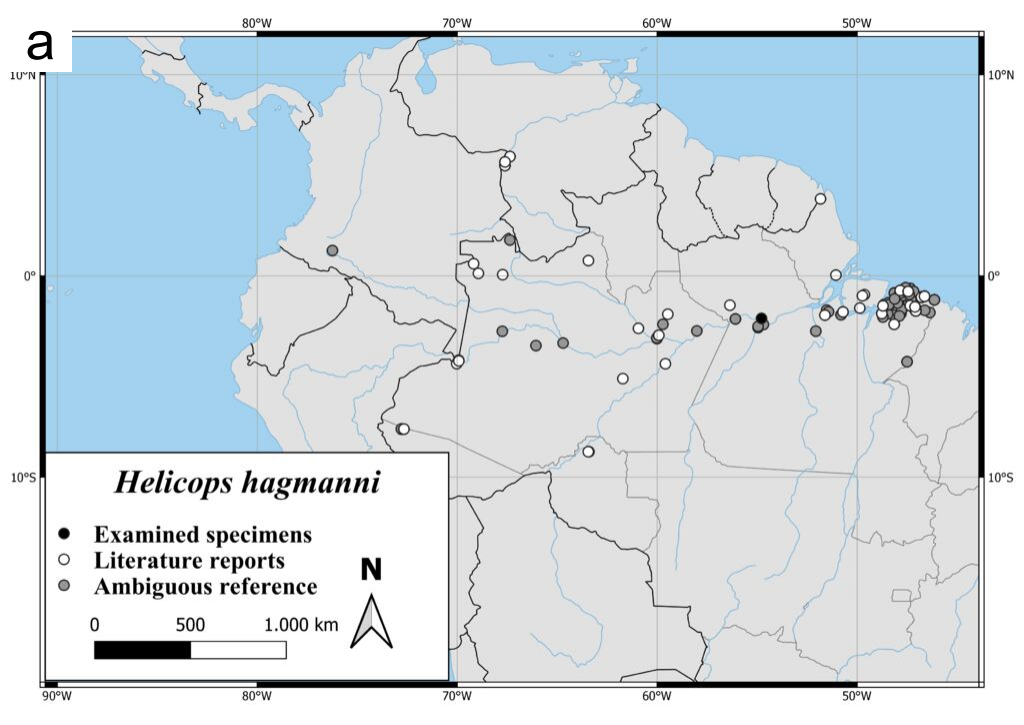


**Figure 2b.**
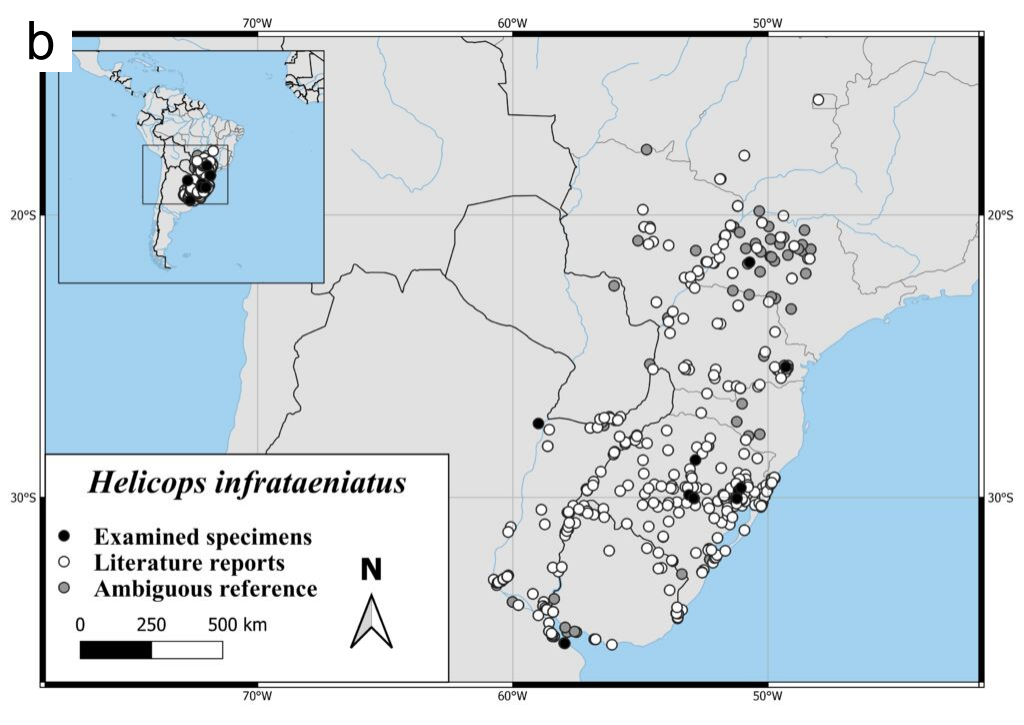


**Figure 2c.**
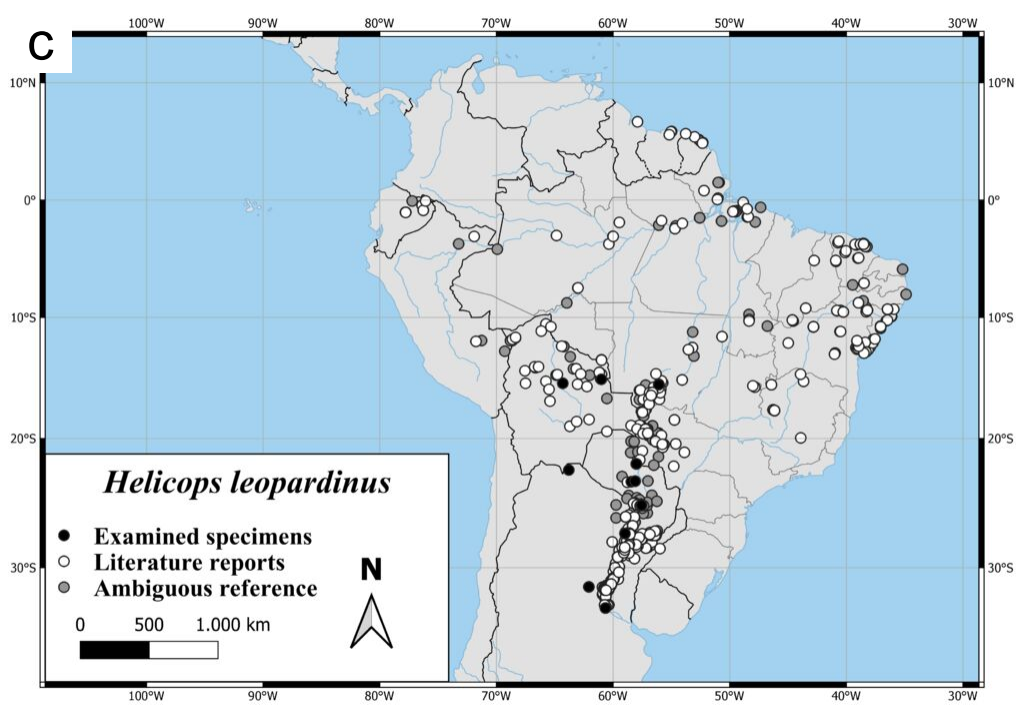


**Figure 2d.**
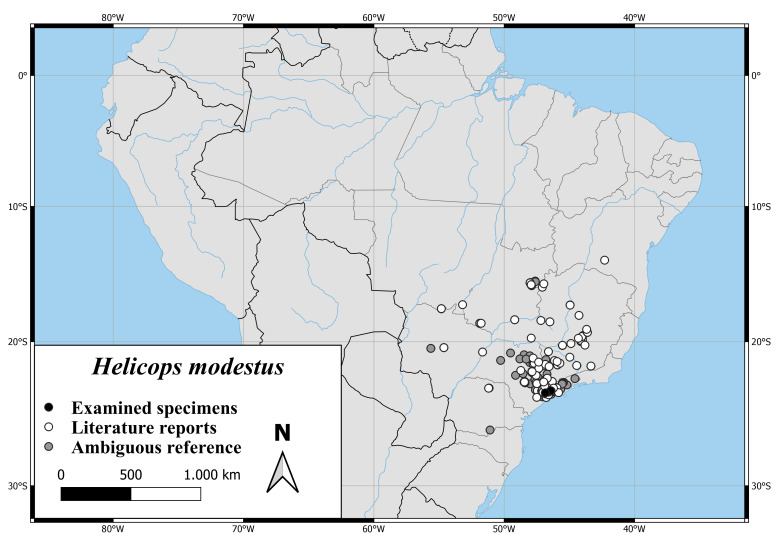


**Figure 2e.**
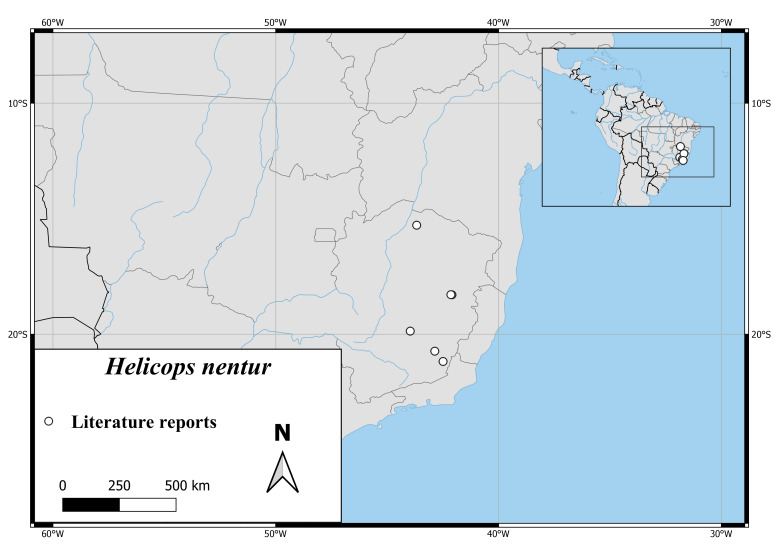


**Figure 2f.**
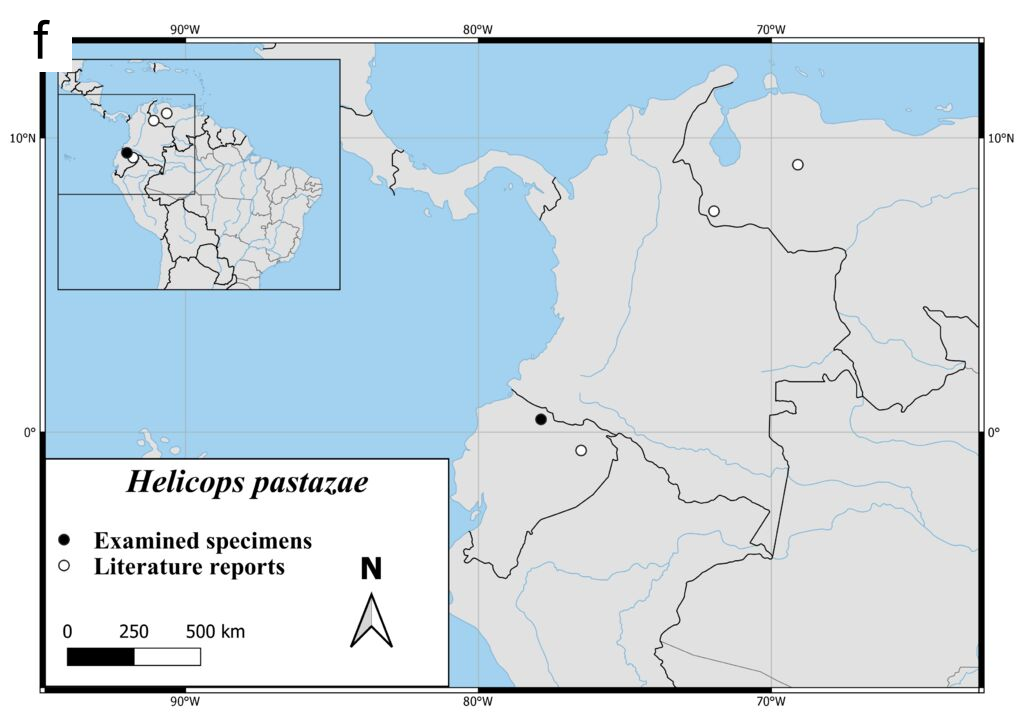


**Figure 3a.**
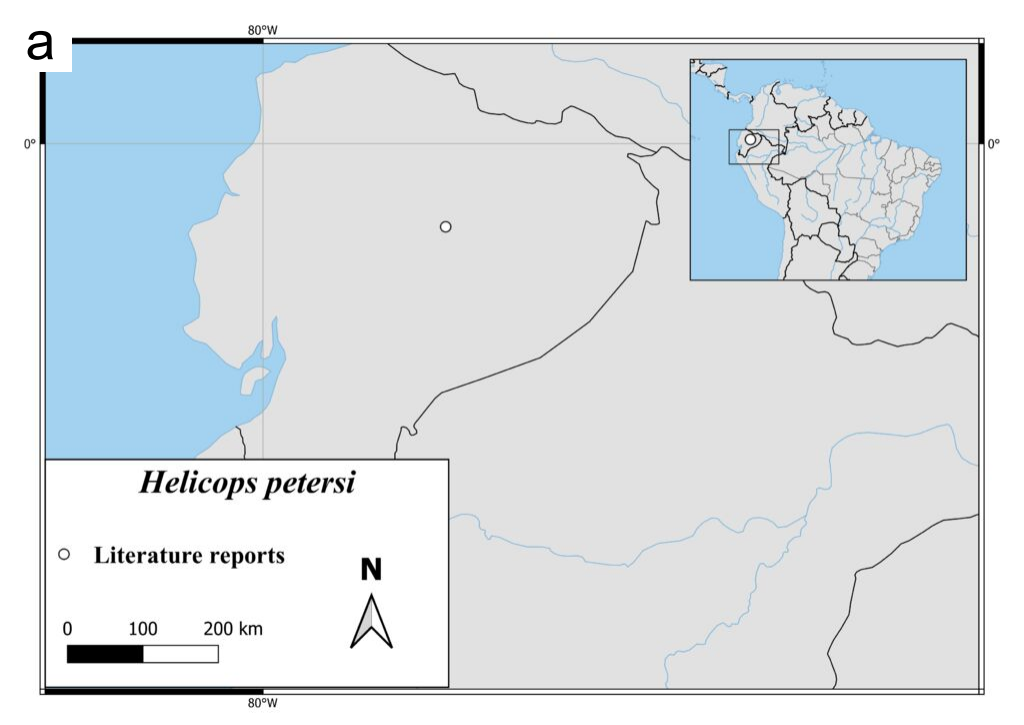


**Figure 3b.**
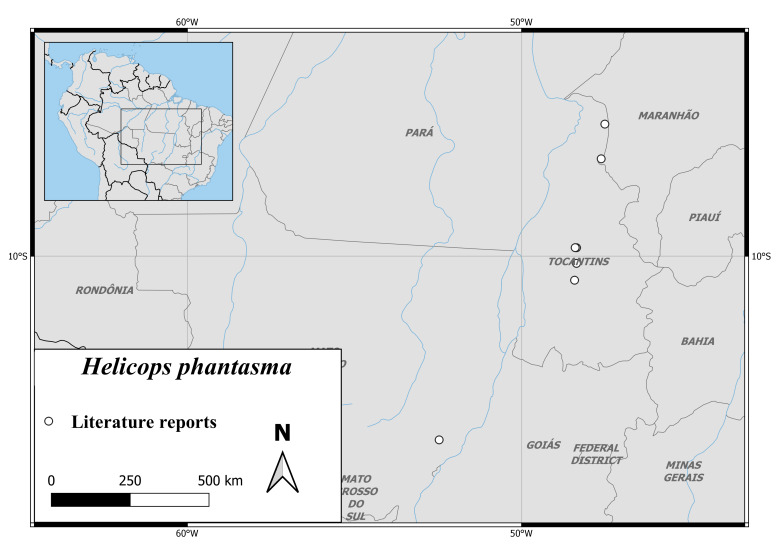


**Figure 3c.**
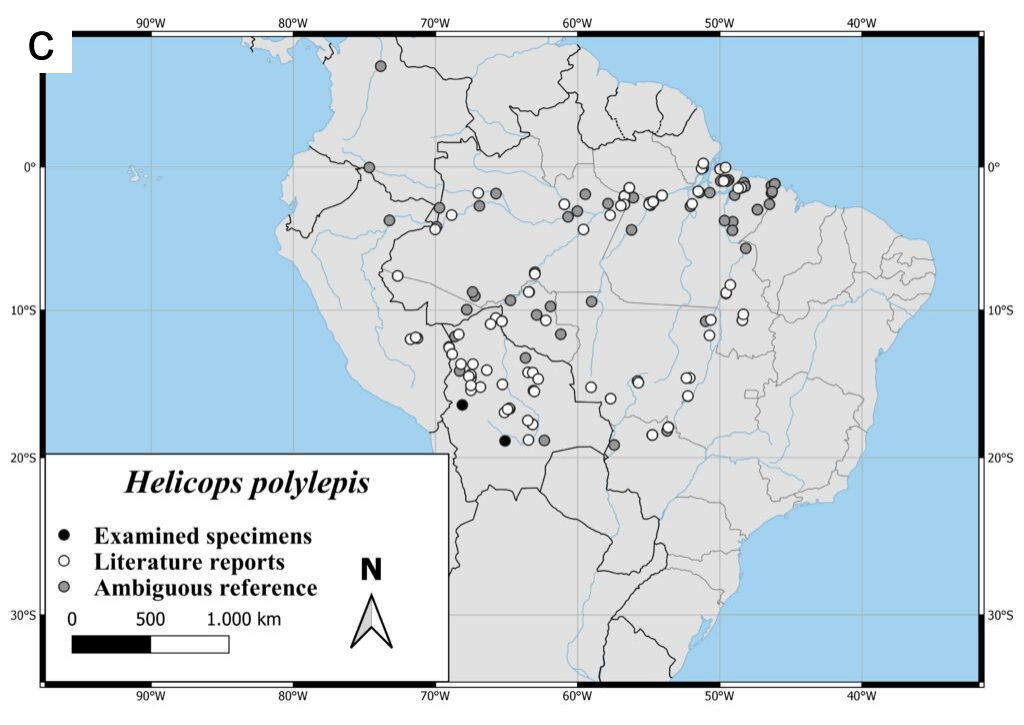


**Figure 3d.**
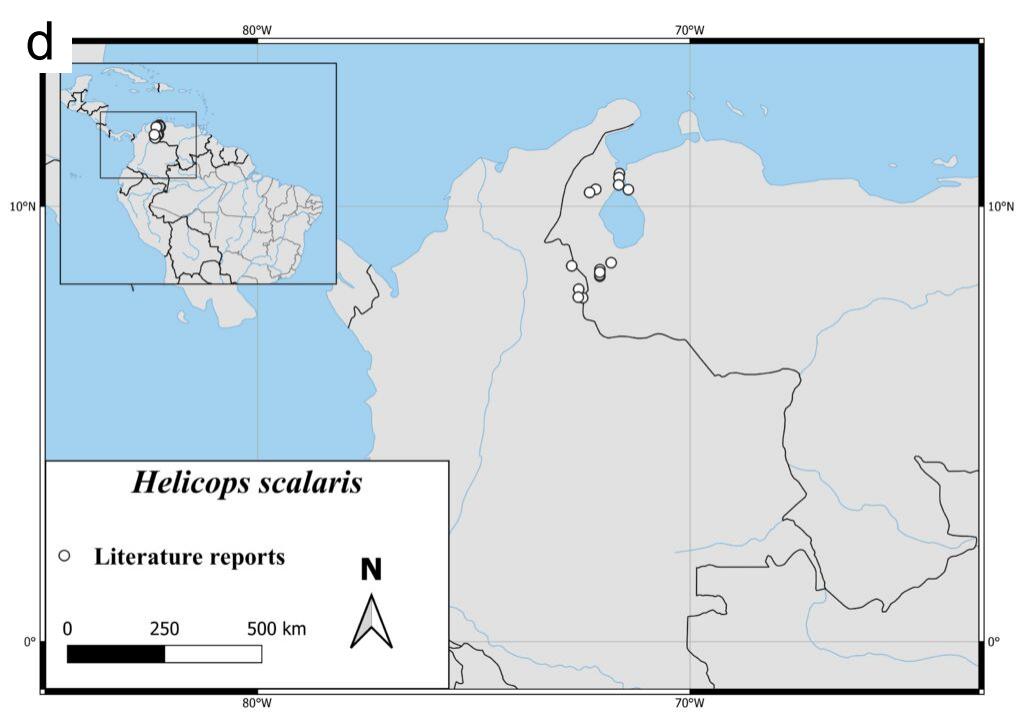


**Figure 3e.**
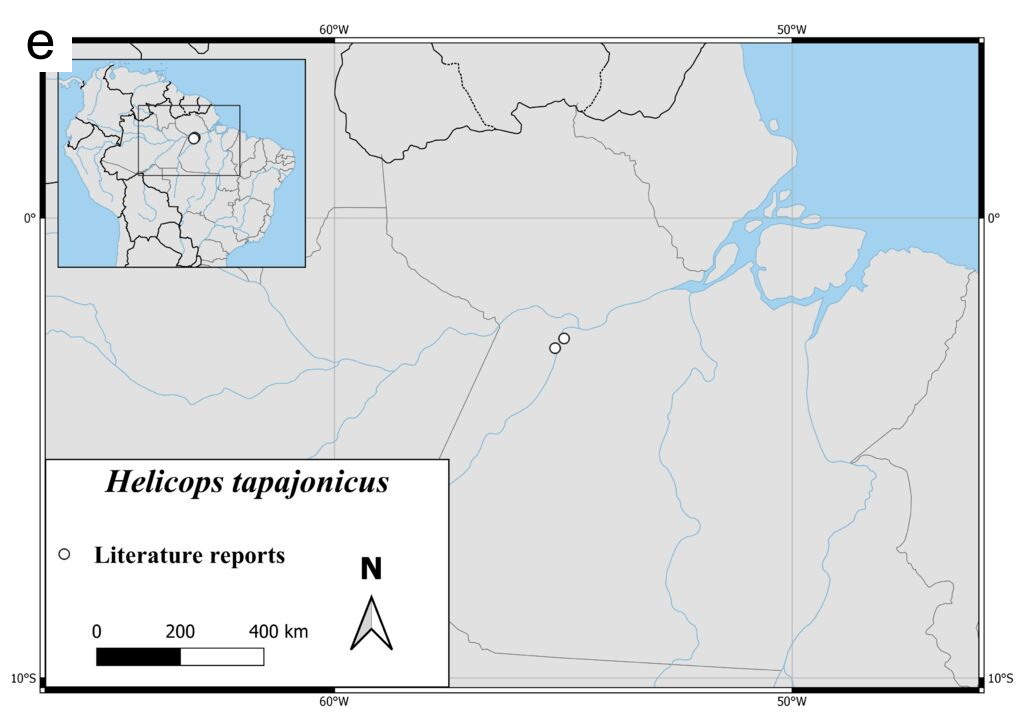


**Figure 3f.**
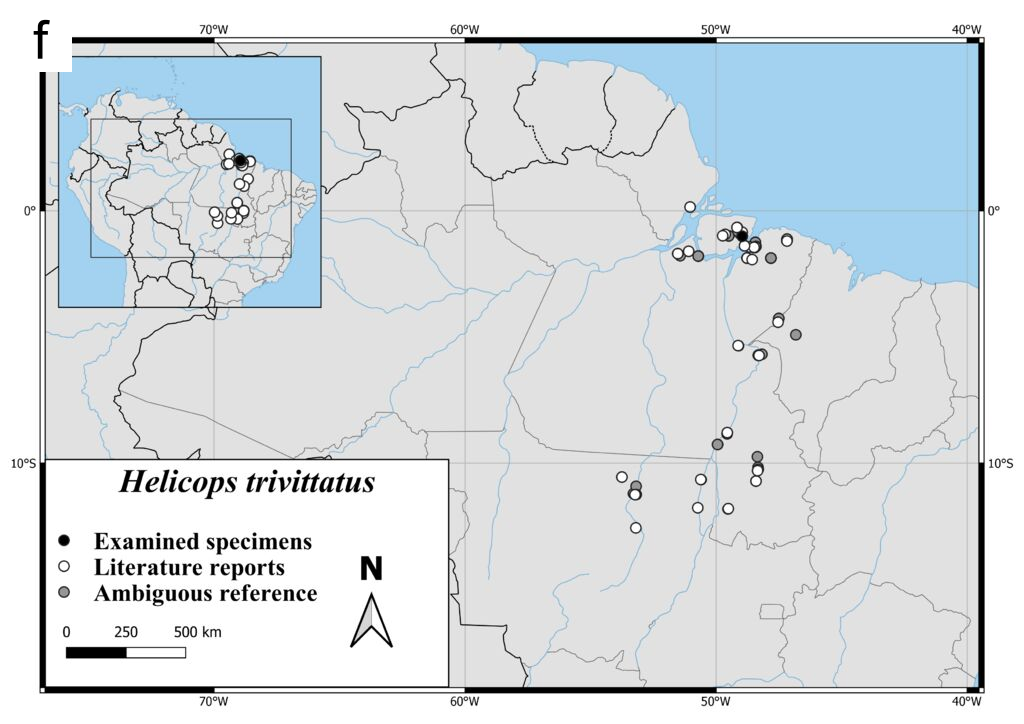


**Figure 4.**
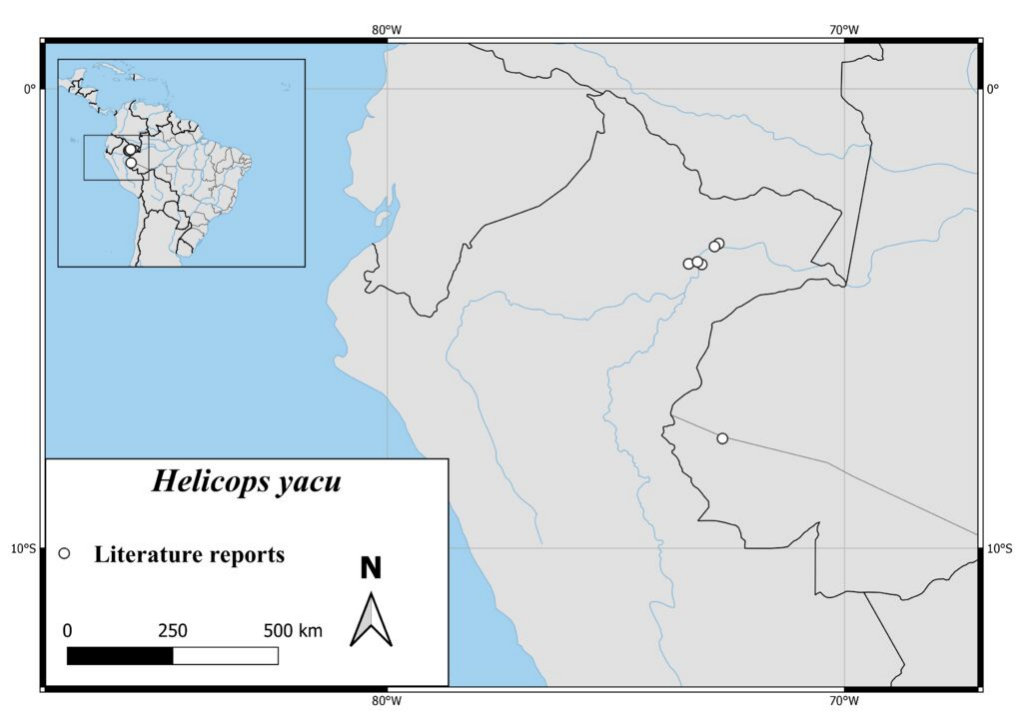
Distribution of *Helicopsyacu*. White circles represent literature reports. For the coordinates and references of the distribution points from literature, see Suppl. material 2.

**Table 1.** Summarised results of the morphologic examination of 190 specimens. Abbreviations: N: Number of examined individuals; SVL: snout-vent length; TL: tail length; VE: ventrals; SC: subcaudals; SCK: presence of subcaudal keels; LO: loreals; PRO: preoculars; PSO: postoculars; AT: anterior temporals; PT: posterior temporals; SL: supralabials; SL+E: supralabials in contact with the eye; IL: infralabials; DSM: dorsal scale rows at mid-body; DKM: dorsal keels at mid-body; DSP: dorsal scale rows at posterior body; DKP: dorsal keels at posterior body; CL: cloacal plate; div: divided; IG: intergenials; NA: if nasal is divided; sdiv: semi-divided.; values in brackets show observations we rate as natural abnormalities, which are discussed in the respective species account. We rounded values to the third decimal place, lengths are in millimetres. See Suppl. material 1 for data of all specimens.

	*H.angulatus*	*H.carinicaudus*	*H.danieli*	*H.hagmanni*	*H.infrataeniatus*
N	47	11	5	2	57
SVL ♂	229–420	414–570	409	460	157–489
SVL ♀	145–680	280–810	163–620	575	136–600
TL ♂	128–275	160–190	194	174	66–174
TL ♀	30–325	90–203	62–185	185	45–194
TL/SVL ♂	0.417–0.696	0.330–0.387	0.474	0.378	0.311–0.526
TL/SVL ♀	0.185–0.922	0.235–0.321	0.271–0.380	0.323	0.209–0.462
VE ♂	103–119	139–141	128	123	114–128
VE ♀	104–125 (130; 156)	128–146	131–139	131	113–130
SC ♂	69–100	64–71	79	59	58–88
SC ♀	58–92	51–59	60–81	50	49–74
SCK	present	absent	absent	present	absent (1x present)
LO	1	1–2	1	1	0–3
PRO	1–2	1–2	1	1	1–2
PSO	2–3	2	2	1–2	2
AT	1–3	1	1	1	1–2
PT	2–4	1–2	2–3	3	1–3
SL	8–9	7–8	7–8	8	7–8
SL+E	IV	IV, III–IV	IV, IV–V	IV	III, III–IV, IV
IL	9–11	9–10	9–12	11–12	9–12
DSM	17–20	17–19	18–19	25–27	17–20
DSM	present	present	present	present	present
DSP	16-17	17	17	21	15–17
DKP	present	present	present	present	present
CL	div	div	div	div	div
IG	absent	absent	absent	present	absent
NA	sdiv	sdiv	sdiv	sdiv	sdiv

**Table 2.** See description Table 1.

	*H.leopardinus*	*H.modestus*	*H.pastazae*	*H.polylepis*	*H.trivittatus*
N	44	12	1	7	4
SVL ♂	217–495	165–305	415	170–235	328
SVL ♀	139–620	98–438		149–407	195–356
TL ♂	114–194	68–125	250	86–98	118
TL ♀	53–235	33–129		58–69	118–134
TL/SVL ♂	0.333–0.547	0.410–0.412	0.602	0.417–0.506	0,360
TL/SVL ♀	0.313–0.557	0.295–0.432		0.219–0.463	0.376–0.400
VE ♂	110–129	114–116	130	123–126	122
VE ♀	109–127	112–124		122–128	116–119
SC ♂	53–88	64–67	108	72–101	53
SC ♀	56–88 (109)	49–70		78–88	62–67
SCK	absent	absent	present	absent	absent
LO	0–2	0–2	1	1	1
PRO	1–2	1–2	1	1	2
PSO	1–2	2	2	2	2
AT	1–2	1	1	1–2	1
PT	1–3	2	2	2–4	2
SL	7–9	7–8	7	7–8	8
SL+E	III–IV, IV, IV-V	III–IV, IV	IV	III–IV, IV	IV
IL	9–11	9–11	10–11	10–13	11–14
DSM	18–19	17–20	23	23	23
DSM	present	present	present	present	present
DSP	16–19	15–19	16	19–21	19
DKP	present	present	present	present	present
CL	div	div	div	div	div
IG	absent	absent	present	absent	variable
NA	sdiv	sdiv	sdiv	sdiv	sdiv

**Table 3.** Pholidosis characters of the female specimen SMF 34035. Abbreviations: SVL: snout-vent length; TL: tail length; VE: ventrals; SC: subcaudals; presence of subcaudal keels (SCK); PRO: preoculars; PSO: postoculars; LO: loreal; AT: anterior temporals; NA: nasal; PT: posterior temporals; SL: supralabials; SL+E: supralabials in contact with the eye; IL: infralabials; DSM: dorsal scale rows at mid-body; DKM: dorsal keels at mid-body; DSP: dorsal scale rows at posterior body; DKP: dorsal keels at posterior body; CL: cloacal plate; IG: presence of Intergenials; Decimal values were rounded to the third decimal place, lengths are in millimetres. See Suppl. material 1 for data of all specimens.

SVL	365	PT right	2
TL	189	PT left	2
TL/SVL	0,518	SL right	8
VE	124	SL left	8
SC	75	SL+E right	IV
SCK	absent	SL+E left	IV
PRO right	1	IL right	10
PRO left	1	IL left	10
LO right	1	DSM	17
LO left	1	DKM	present
PSO right	2	DSP	17
PSO left	2	DKP	present
AT right	1	CL	divided
AT left	1	IG	absent
NA	semi-divided		

## References

[B6769722] Achaval Elena Federico (2001). Systematic update and maps of distribution of the reptiles of Uruguay. Actualizacion sistematica y mapas de distribucion de los reptiles del Uruguay. Smithsonian Herpetological Information Service.

[B6769758] Carreira Vidal Santiago, Meneghel Melitta, Achaval Federico (2005). Reptiles de Uruguay.

[B6769766] Costa Henrique C., Santana Diego J., Leal Fernando, Koroiva Ricardo, Garcia Paulo C. A. (2016). A new species of *Helicops* (Serpentes: Dipsadidae: Hydropsini) from southeastern Brazil. Herpetologica.

[B7341460] Deiques C. H., Cechin S. Z. (1991). O status de *Helicopscarinicaudus* (Wied, 1825)(Serpentes: Colubridae). Acta Biológica Leopoldensia.

[B6769785] Di Pietro Diego Omar, Alcalde Leandro, Williams Jorge Daniel (2014). Nasal cartilages, hyobranchial apparatus, larynx, and glottal tubes in four species of Hydropsini (Serpentes: Dipsadidae: Xenodontinae). Vertebrate Zoology.

[B6769794] Dowling Herndon G. (1951). A proposed standard system of counting ventrals in snakes. British Journal of Herpetology.

[B6769821] França Rafaela Cândido de, Germano Carlos Eduardo de Souza, França Frederico Gustavo Rodrigues (2012). Composição de uma taxocenose de serpentes em uma área urbana na Mata Atlântica da Paraíba, Nordeste do Brasil. Copeia.

[B7351909] Giraudo Alejandro (2001). Serpientes de la Selva Paranaense y del Chaco Húmedo.

[B6769847] Hernández-Ruiz Emil José, Wariss Figueiredo Manoela, Brito Pezzuti Juarez Carlos (2014). Bycatch of *Helicopsangulatus* (Linnaeus, 1758) (Reptilia: Squamata: Colubridae) in hoop-traps used to capture fresh water turtles on the coast of Pará, Brazil. Acta Biológica Colombiana.

[B6769856] Kawashita-Ribeiro Ricardo Alexandre, Ávila Robson Waldemar, Morais Drausio Honorio (2013). A new snake of the genus *Helicops* Wagler, 1830 (Dipsadidae, Xenodontinae) from Brazil. Herpetologica.

[B6769865] Koski D. A., Monico A. T., Koski A. P.V. (2016). *Helicopsangulatus* (Brown-banded Watersnake). Predation. Herpetological Review.

[B6769874] Linnaeus Carolus (1758). Systema naturae (Systema naturae per regna tria naturae, secundum classes, ordines, genera, species, cum characteribus, differentiis, synonymis, locis. Tomus I. Editio decima, reformata).

[B6769882] Moraes-da-Silva Antonio, Amaro Renata Cecilia, Nunes Sales Pedro M., Strüssmann Christine, Teixeira Junior Mauro, Andrade Albedi, J., Sudre Vinicius, Recoder Renato, Rodrigues Trefaut Miguel, Curcio Felipe Franco (2019). Chance, luck and a fortunate finding: a new species of watersnake of the genus *Helicops* Wagler, 1828 (Serpentes: Xenodontinae), from the Brazilian Pantanal wetlands. Zootaxa.

[B6769897] Moraes-da-Silva Antonio, Amaro Renata Cecília, Nunes Pedro M. Sales, Rodrigues Miguel Trefaut, Curcio Felipe Franco (2021). Long known, brand new, and possibly threatened: a new species of watersnake of the genus *Helicops* Wagler, 1828 (Serpentes; Xenodontinae) from the Tocantins-Araguaia River Basin, Brazil. Zootaxa.

[B7347013] Murphy John C, Muñoz-Mérida Antonio, Auguste Renoir J., Lasso-Alcala Oscar, Rivas Gilson A, Jowers Michael J. (2020). Evidence for cryptic diversity in the Neotropical water snake, Helicopsangulatus (Linnaeus, 1758)(Dipsadidae, Hydropsini), with comments on its ecology, facultative reproductive mode, and conservation. Amphibian & Reptile Conservation.

[B7341469] Nogueira Cristiano C., Argôlo Antonio J. S., Arzamendia Vanesa, Azevedo Josué A., Barbo Fausto E., Bérnils Renato S., Bolochio Bruna E., Borges-Martins Marcio, Brasil-Godinho Marcela, Braz Henrique, Buononato Marcus A., Cisneros-Heredia Diego F., Colli Guarino R., Costa Henrique C., Franco Francisco L., Giraudo Alejandro, Gonzalez Rodrigo C., Guedes Thaís, Hoogmoed Marinus S., Marques Otavio A. V., Montingelli Giovanna G., Passos Paulo, Prudente Ana L. C., Rivas Gilson A., Sanchez Paola M., Serrano Filipe C., Silva Nelson J., Strüssmann Christine, Vieira-Alencar João Paulo S., Zaher Hussam, Sawaya Ricardo J., Martins Marcio (2019). Atlas of Brazilian Snakes: Verified Point-Locality Maps to Mitigate the Wallacean Shortfall in a Megadiverse Snake Fauna. South American Journal of Herpetology.

[B6769907] Peters J. A., Orejas-Miranda B. (1970). Catalogue of the Neotropical Squamata: Part I. Snakes. United States National Museum Bulletin.

[B7495187] Rossman D. A. (1975). Redescription of the South American colubrid snake Helicopshagmanni Roux. Herpetologica.

[B6769925] Rossman Douglas Athon, Dixon James Ray (1975). A new colubrid snake of the genus *Helicops* from Peru. Herpetologica.

[B6769943] Rossman Douglas Athon, Abe Augusto Shinya (1979). Comments on the taxonomic status of *Helicopsyacu* (Serpentes: Colubridae). Proceedings of the Louisiana Academy of Sciences.

[B6769961] Rossman Douglas Athon (2002). Variation in the xenodontid water snake *Helicopsscalaris* Jan, and the status of *H.hogei* Lancini. Occasional Papers of the Museum of Natural Science.

[B6770023] Vaz-Silva W., Oliveira RM., Gonzaga AFN., Pinto KC., Poli FC., Bilce TM., Penhacek M., Wronski L., Martins JX., Junqueira TG., Cesca LCC., Guimarães VY., Pinheiro RD. (2015). Contribuições para o conhecimento de anfíbios e répteis da Volta Grande do Xingu, norte do Brasil. Brazilian Journal of Biology.

[B7574594] Wagler J. G. (1830). Natürliches System der Amphibien: mit vorangehender Classification der Säugethiere und Vögel: ein Beitrag zur vergleichenden Zoologie.

[B6838983] Whitworth Andrew, Beirne Christopher (2011). Reptiles of the Yachana Reserve.

[B6770066] Yuki R. N., Castano Olga Victoria (1998). Geographic distribution note of water-snake *Helicopsdanieli* Amaral, 1937 (Colubridae: Xenodontinae). The Snake.

